# A New Class of Electronic Devices Based on Flexible Porous Substrates

**DOI:** 10.1002/advs.202105084

**Published:** 2022-01-17

**Authors:** Yiyuan Zhang, Tengyuan Zhang, Zhandong Huang, Jun Yang

**Affiliations:** ^1^ Department of Mechanical and Materials Engineering University of Western Ontario London ON N6A 5B9 Canada; ^2^ Shenzhen Institute for Advanced Study University of Electronic Science and Technology of China Shenzhen 518000 P. R. China

**Keywords:** biocompatibility, breathability, deformability, electronic device, flexible porous substrate, pore structure

## Abstract

With the advent of the Internet of Things era, the connection between electronic devices and humans is getting closer and closer. New‐concept electronic devices including e‐skins, nanogenerators, brain–machine interfaces, and implantable medical devices, can work on or inside human bodies, calling for wearing comfort, super flexibility, biodegradability, and stability under complex deformations. However, conventional electronics based on metal and plastic substrates cannot effectively meet these new application requirements. Therefore, a series of advanced electronic devices based on flexible porous substrates (e.g., paper, fabric, electrospun nanofibers, wood, and elastic polymer sponge) is being developed to address these challenges by virtue of their superior biocompatibility, breathability, deformability, and robustness. The porous structure of these substrates can not only improve device performance but also enable new functions, but due to their wide variety, choosing the right porous substrate is crucial for preparing high‐performance electronics for specific applications. Herein, the properties of different flexible porous substrates are summarized and their basic principles of design, manufacture, and use are highlighted. Subsequently, various functionalization methods of these porous substrates are briefly introduced and compared. Then, the latest advances in flexible porous substrate‐based electronics are demonstrated. Finally, the remaining challenges and future directions are discussed.

## Graphical abstract

The progress of various flexible porous substrate‐based electronics is systematically reviewed with a focus on the role and impact of porous structure on device performance. Different functionalization methods and advanced electronic devices on flexible porous substrates are demonstrated. The key challenges and future directions for developing a new generation of electronics on flexible porous substrates are also proposed.

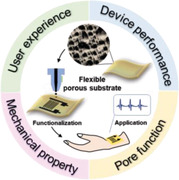

## 1 Introduction

As the cornerstone of the upcoming Internet of Things era, electronic devices are experiencing tremendous changes to meet new application demands. They are evolving from portable to wearable or implantable, from impermeable to breathable, from environmentally unfriendly to biodegradable, from rechargeable to self‐powered. To achieve these features, their substrates are no longer limited to traditional metal and plastic. In recent years, a new class of electronic devices based on flexible porous substrates are receiving increasing attention for their potential revolutionary applications in soft robots,^[^ ^1^ ^]^ e‐skins,^[^ ^2^ ^]^ brain–machine interfaces,^[^ ^3^ ^]^ implantable bioelectronics,^[^ ^4^ ^]^ and sustainable energy harvest^[^ ^5^ ^]^ and storage.^[^ ^6^ ^]^ Numerous flexible porous materials have been explored as substrates for next‐generation electronics to accommodate new working environments and requirements. For example, paper‐based electronics^[^ ^7^ ^]^ have been widely investigated for low‐cost, disposable, and portable electronics. To achieve a new interactive experience between electronic devices and humans, wearable electronics that are conformable, breathable, and lightweight have been successfully fabricated using fabrics^[^ ^8^ ^]^ and electrospun nanofibers.^[^ ^9^ ^]^ In addition, more and more electronic devices are integrated into natural biodegradable materials^[^ ^10^ ^]^ such as wood, leaves, natural silk, and animal skin to reduce the environmental impact of e‐waste. Porous elastic polymers such as polydimethylsiloxane (PDMS)^[^ ^11^ ^]^ and polyurethane (PU)^[^ ^12^ ^]^ have been exploited to construct multidirectional stretchable electronics, which can maintain their functions under big deformations. In the traditional sense, the porous structure and rough surface of these substrates are unfavorable for electronic manufacturing because they are incompatible with well‐established silicon‐based electronics processing technologies. However, thanks to the diversified development of functionalization techniques and conductive materials, these porous structures have turned from inferior to superior in various applications. To name a few, the porous structure can enhance the carrier transport efficiency of energy storage devices.^[^ ^6^ ^]^ The porous substrate can strengthen the adhesion of the sensing material, thereby improving the sensitivity of the sensor.^[^ ^10^ ^]^ Meanwhile, it offers ample deformation space to eliminate the stress concentration of the active material, thus preventing crack propagation and ensuring high damage resistance.^[^ ^11^ ^]^

**Figure 1:**
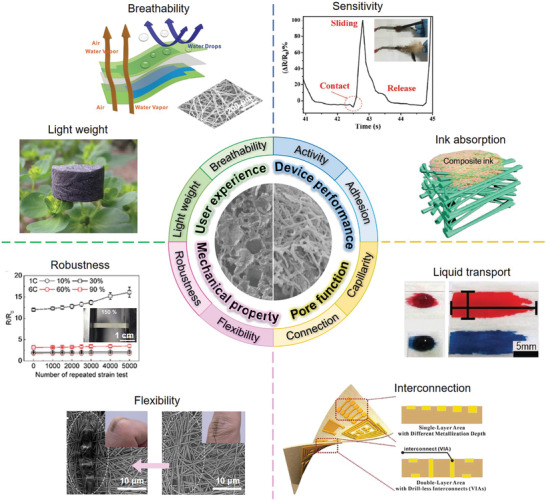
The advantages of flexible porous substrates for electronic products in terms of user experience, device performance, mechanical property, and pore function. “Light weight”: Reproduced with permission.^[^ ^13^ ^]^ Copyright 2017, The Royal Society of Chemistry. “Breathability”: Reproduced with permission.^[^ ^14^ ^]^ Copyright 2017, Wiley‐VCH. “Sensitivity”: Reproduced with permission.^[^ ^15^ ^]^ Copyright 2018, Wiley‐VCH. “Ink absorption”: Reproduced with permission.^[^ ^16^ ^]^ Copyright 2018, Wiley‐VCH. “Robustness”: Reproduced with permission.^[^ ^19^ ^]^ Copyright 2017, Wiley‐VCH. “Flexibility”: Reproduced with permission.^[^ ^20^ ^]^ Copyright 2017, Springer Nature. “Interconnection”: Reproduced with permission.^[^ ^18^ ^]^ Copyright 2018, Wiley‐VCH. “Liquid transport”: Reproduced with permission.^[^ ^21^ ^]^ Copyright 2019, Wiley‐VCH.

The general characteristics of flexible porous materials include low density, large specific surface area, strong deformability, abundant material exchange channels, and enhanced chemical activity. When constructing electronic devices on porous substrates, these characteristics can boost device performance from different perspectives as shown in **Figure** **1**. i) From the user experience, the lightweight porous substrate facilitates the manufacture of portable electronic devices.^[^ ^13^ ^]^ Meanwhile, the pore structure provides a large number of free passages for air and water vapor, making the substrate breathable and comfortable for long‐term wearing.^[^ ^14^ ^]^ ii) In terms of device performances, the porous substrate affords a large interfacial area and sufficient bonding points for the active materials, thereby enhancing their activity^[^ ^15^ ^]^ and adhesion.^[^ ^16^ ^]^ In addition, big porosity can store more conductive materials, enhance conductivity, and electrochemical performance, thus promoting sensitivity^[^ ^17^ ^]^ and energy conversion efficiency.^[^ ^18^ ^]^ iii) Mechanically, the 3D porous structure endows the substrate with sufficient space for deformation, which can be bent, stretched, folded, sheared, and twisted, exhibiting outstanding robustness^[^ ^19^ ^]^ and flexibility.^[^ ^20^ ^]^ iv) The pore structures enable some unique functions. For example, the interconnected pore channels allow the electronic components on different layers to be directly connected, eliminating the need for drilling holes or complicated external circuits.^[^ ^18^ ^]^ Therefore, both sides of the porous substrate can be functionalized and easily integrated. The capillarity of porous substrates can be used for fluid transport,^[^ ^21^ ^]^ fluidic valves,^[^ ^22^ ^]^ fluidic delay switch,^[^ ^23^ ^]^ without the need for an external pump. In addition, capillarity benefits the rapid absorption and penetration of active material and alleviates uneven evaporation and nonuniform pattern caused by the coffee stain effect during printing or coating.^[^ ^24^ ^]^

In previous reviews of electronics based on flexible porous substrates, the authors usually only focus on one type of porous substrate. For example, paper‐based low‐cost electronics,^[^ ^6^, ^25^ ^]^ fabric‐based wearable electronics,^[^ ^26^ ^]^ electrospun nanofiber‐based flexible electronics,^[^ ^27^ ^]^ and wood‐based green electronics^[^ ^28^ ^]^ have been extensively reviewed by different groups. However, these articles mainly concentrate on the device fabrication methods, functional materials, and device applications, few reviews pay attention to the role of pore structures of porous substrates and their impact on device performance. In addition, there is still a lack of comprehensive comparison and summary of the performance of different porous substrates. In this review, we will focus on the latest progress in flexible porous substrate‐based electronics, where pore structures are used as advantages to improve device performance and realize new functions. We hope this review may provide timely references for the development of high‐performance, user‐friendly electronic devices on flexible porous substrates for different applications. We first introduce different types of flexible porous substrates including their unique properties, manufacturing, classification, advantages, and disadvantages. Subsequently, diverse functionalization techniques for flexible porous substrates are expounded. Next, we demonstrated various advanced electronic devices based on flexible porous substrates with novel functions. Lastly, the scientific and technical challenges and future directions for developing a new generation of electronics on flexible porous substrates are proposed.

## 2 Flexible Porous Substrate Materials

**Figure 2:**
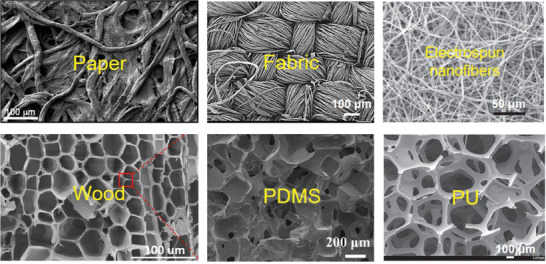
The micromorphology of the typical flexible porous substrates. “Paper”: Reproduced with permission.^[^ ^29^ ^]^ Copyright 2018, Wiley‐VCH. “Fabric”: Reproduced with permission.^[^ ^30^ ^]^ Copyright 2018, Elsevier. “Electrospun nanofibers”: Reproduced with permission.^[^ ^31^ ^]^ Copyright 2014, Wiley‐VCH. “Wood”: Reproduced with permission.^[^ ^32^ ^]^ Copyright 2020, American Chemical Society. “PDMS”: Reproduced with permission.^[^ ^33^ ^]^ Copyright 2021, Springer Nature. “PU”: Reproduced with permission.^[^ ^34^ ^]^ Copyright 2016, Wiley‐VCH.

Substrate materials play a vital role in the overall performance of electronic devices. They not only provide mechanical support but also can serve as functional components of the constructed devices. Currently, there are two ways to fabricate electronic devices on flexible porous substrates with rough surfaces. One is to fill the pores with a coating layer to make the surface smooth before making electronic components. The other is to manufacture electronic devices directly on porous substrates. Here, we mainly focus on the latter method, in which the unique properties of porous substrates are retained and utilized. According to the different pore structures, the porous substrate is divided into fiber‐based and non‐fiber‐based. Fiber‐based porous substrates are composed of fiber raw materials and usually have excellent fatigue resistance. Non‐fiber‐based porous substrates mainly refer to porous elastomers, which exhibit superior stretchability. **Figure** **2** demonstrates the micromorphology of the typical fiber‐based and non‐fiber‐based flexible porous substrates. Different compositions and pore structures endow these porous materials with distinct physical and chemical properties. Hence, electronic devices built on them can be applied to different application scenarios, and their porous structure can help devices achieve specific functions. To compare different flexible porous substrate materials intuitively, their remarkable features (e.g., porosity, breathability, deformability), advantages, disadvantages, and typical devices are summarized in **Table** **1**.

**Table 1:** Remarkable features and typical devices of various flexible porous substrates

Substrate	Advantages	Disadvantages	Remarkable properties	Typical devices
Paper	Renewable, biodegradable, combustible, flexible, lightweight, low‐cost	Susceptible to humidity	Capacitance retention (80% after 10 000 charging/discharging cycles with current density of 20 mA cm^−2^)	Supercapacitor^[^ ^35^ ^]^
			Adding microfluidic functions to conventional circuits	Electrofluidics^[^ ^36^ ^]^
			Hygroexpansive electrothermal actuation, bending, and accordion type motions	Actuator^[^ ^37^ ^]^
			Air permeability (333 mm s^−1^), wet stability (85% voltage retention after 4 soaking cycles)	Triboelectric nanogenerator ^[^ ^38^ ^]^
Fabrics	Light weight, big porosity, softness, comfort, warmth retention, breathability, good elasticity and recovery ability, durability, washability, sewability	Crack or delamination between functional materials and substrate	High permeability for trans epidermal water loss (≈10 gh^−1^m^−2^ at a thickness of 100 mm), elasticity (able to accommodate ≈250% tensile strain)	Stretchable electronics^[^ ^39^ ^]^
			Foldability (180°, 30 times)	Self‐powered textile^[^ ^40^ ^]^
			Moisture permeation (0.929 mg cm^−2^·h^−1^), pressure detection sensitivity (4.46 kPa^−1^), response time (≈39 ms), mechanical stability (10 000 cycles of bending or compression)	Pressure sensor^[^ ^41^ ^]^
			Ammonia gas response sensitivity: 10.3 for 50 ppm, pressure response sensitivity: 0.073 kPa^−1^, temperature response sensitivity: 0.44 °C^−1^)	Multifunctional sensor^[^ ^42^ ^]^
Electrospun nanofibers	Lightweight, transparent, biocompatible, breathable, flexible, washable, stitchable, enhanced surface area, controllable porosity, customized fiber structures	Difficulty in the production of highly aligned nanofibers over a large area, slow spinning speed, small volume output	Air permeability (Gurley value = 17.3 s/100 mL), pressure detection sensitivity (4.2 kPa^−1^), response time (<26 ms), detection limit (1.6 Pa)	Pressure sensor^[^ ^14^ ^]^
			Tensile modulus (89.8 MPa), break elongation (5.89%), capacitance retention (85.6% after 20 000 cycles), bending stability (500 cycles of 180°)	Supercapacitor^[^ ^43^ ^]^
			Substrate porosity (71.2%), flexibility, and mechanical stability (1000 bending cycles with a 5 mm bending radius), on‐demand antibiotic release	On‐demand therapy^[^ ^44^ ^]^
			The tensile force exerted by the nanofiber substrate on the cardiomyocyte sheet (<0.4 mN or less with a width of 20 mm at a strain of 10%), no interference to cardiomyocytes	Ultrasoft electronics^[^ ^45^ ^]^
Natural porous materials	Fully biodegradable, biocompatible, renewable, low‐cost, lightweight, abundant functional groups, selective adsorption, antimicrobial	Complex structure–property relationships between substrates and device performances	Water‐vapor permeability (3714 g m^−2^ d^−1^), fully degradable, strain detection sensitivity (0.144 kPa^−1^), response time (200 ms), operational stability (loading/unloading pressure of 1.3 kPa for 15 000 cycles)	Sensory skin^[^ ^46^ ^]^
			Hydroscopicity, ionic conductivity, humidity sensing range (0–90% RH), response time (≈1.99 s)	Organic humidity sensor^[^ ^47^ ^]^
			FDA approved, transparency, elastic modulus (2.04 ± 0.26 GPa), failure strain (32%)	Implantable electronics^[^ ^48^ ^]^
			Transport sweat (>11.25 mm s^−1^ at the first second)	Guiding layer for on‐skin electronics^[^ ^21^ ^]^
Porous PDMS	Stable chemical, thermal, electrical properties, transparency, biocompatibility, outstanding deformability, good moldability	Difficulty in the production of micropores (<100 nm) and hierarchical porous structure	Pressure detection sensitivity (0.63 kPa^−1^), durability, and stability of the pressure sensor (>10 000 loading and unloading cycles, with a 0.5 n load)	Pressure sensor^[^ ^17^ ^]^
			Durability (>22 500 loading‐unloading cycles at 100% strain)	Self‐healing sensor^[^ ^49^ ^]^
			Tensile elongation (>80%), bendability (>150°), durability (20 000 stretching cycles under 30% strain)	Stretchable circuit^[^ ^50^ ^]^
Porous PU	Good processability, biocompatibility, chemical resistance, excellent elasticity, high tensile/compressive strength, superb recoverability, durability	Stress relaxation behavior, low pore structure control accuracy	PU foam porosity (90%), density (0.11 g cm^−3^), stable piezoresistive sensing signals at a strain of up to 90%	Piezoresistive sensor^[^ ^13^ ^]^
			Workable strain range (320%), response time (<200 ms), durability (9700 stretching cycles at 100% strain)	Strain sensor^[^ ^51^ ^]^
			Detectable pressure range (91‐16 400 Pa, 0.2–60% strain), response time (<20 ms), reproducibility (>50 000 loading–unloading cycles at 40% strain)	Pressure sensor^[^ ^34^ ^]^

### 2.1 Fiber‐Based Substrate Materials

Fiber is a long and thin thread of material that can be derived from nature or man‐made. Fibers can be randomly interconnected or well‐organized into flexible sheet products such as papers and fabrics. The inter‐fiber voids give the fiber‐based material an intrinsic 3D porous structure and excellent breathability. Countless interconnected fiber structures bring it high flexibility and super fatigue resistance. According to the different manufacturing processes and material sources, fiber‐based substrate materials are grouped into the following four categories.

#### 2.1.1 Paper

The history of paper as substrates for electronic devices can be traced back to the manufacture of flexible circuits for credit cards and biomedical sensors in the late 1960s.^[^ ^7^ ^]^ In recent years, a new surge of interest in paper‐based electronics has risen again due to the urgent need for low‐cost and sustainable flexible electronics. With the rapid upgrading of electronic products, the lifetime of electronics has become shorter and shorter, resulting in countless electronic waste and severe environmental pollution. The renewable, biodegradable, combustible, lightweight, and inexpensive nature of the paper makes it an attractive substrate material for environmentally friendly and once‐use‐and‐throw‐away electronics. With the vigorous development of paper‐based electronics, a variety of functional devices have been successfully manufactured on paper, including transistors,^[^ ^52^ ^]^ sensors,^[^ ^25^, ^53^ ^]^ actuators,^[^ ^37^ ^]^ supercapacitors (SCs),^[^ ^6^ ^]^ microfluidic analytical devices,^[^ ^54^ ^]^ and optoelectronic devices.^[^ ^55^ ^]^

**Figure 3:**
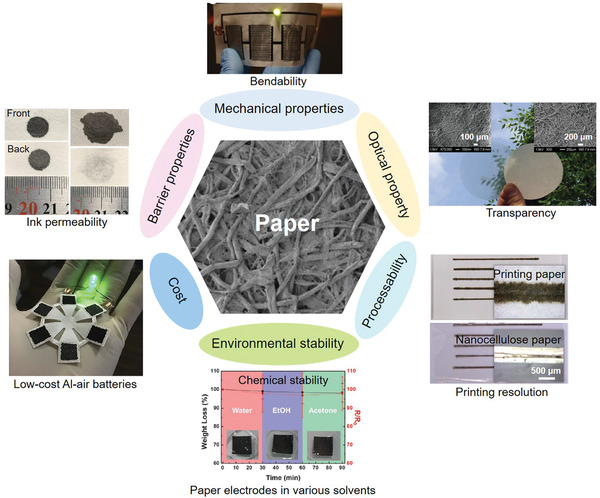
Important criteria for the selection of paper substrates. “Paper”: Reproduced with permission.^[^ ^58^ ^]^ Copyright 2019, The Royal Society of Chemistry. “Ink permeability”: Reproduced with permission.^[^ ^59^ ^]^ Copyright 2019, American Chemical Society. “Bendability”: Reproduced with permission.^[^ ^35^ ^]^ Copyright 2018, Wiley‐VCH. “Transparency”: Reproduced with permission.^[^ ^60^ ^]^ Copyright 2009, Wiley‐VCH. “Printing resolution”: Reproduced with permission.^[^ ^61^ ^]^ Copyright 2019, American Chemical Society. “Paper electrodes in various solvents”: Reproduced with permission.^[^ ^62^ ^]^ Copyright 2016, The Royal Society of Chemistry. “Low‐cost Al‐air batteries”: Reproduced with permission.^[^ ^63^ ^]^ Copyright 2019, Elsevier.

Paper is a cellulose‐rich material that is produced by dewatering the wood‐derived cellulose pulp suspension and pressing the fibers together to form a 3D hierarchical structure. The randomly interconnected cellulose fibers make a porous and rough surface as shown in **Figure** **3**. Currently, there are hundreds of kinds of paper commercially available. Due to the different process parameters, their performance varies greatly. The ink absorbency, hydrophilicity, smoothness, thickness, softness, transparency, and grammage of paper can be easily tuned by changing the pulp composition or applying additional coatings. The common additive agents include mineral fillers (e.g., calcium carbonate, chalk, clays), sizings (e.g., starches, gums, rosins), thickeners (e.g., sodium polyacrylate, carboxymethyl cellulose, starch), and fluorescent whitening agents. In addition, the diameter, length, and physical and chemical properties of cellulose fibers also play a vital role in the transparency and mechanical strength of paper.^[^ ^7^ ^]^

To select the right substrate for a specific application, it is necessary to fully understand the various properties of the substrate material. There are several general selection criteria when choosing different types of porous substrates. Taking paper substrate as an example, six key points that need to be considered are listed in Figure 3. i) Barrier properties. The barrier function of the substrate is the ability to seal oxygen and water vapor to prevent short circuits and degradation. The barrier property of paper depends on its porosity and thickness, which can be improved by applying mineral filler and latex coating.^[^ ^7^ ^]^ ii) Mechanical properties. Mechanical properties of the paper refer to its flexibility, foldability, and bending stiffness associated with the pulp composition and cellulose fiber structure. Generally, paper made of chemical pulp is stronger because of longer fibers. Introducing sizings in the pulp can also strengthen the paper.^[^ ^7^ ^]^ iii) Optical property. Paper made of nanocellulose fibers is highly transparent and can be applied in optoelectronic devices.^[^ ^56^ ^]^ The transparent paper can also be produced by employing transparent materials such as wax or immersing opaque paper in ionic liquids or sulfuric acid.^[^ ^57^ ^]^ iv) Processability. The processability of paper refers to its compatibility with specific functionalization methods. For example, its surface roughness should meet the minimum requirements of the processing technology, and the impurities in the paper should be inert to the processing chemicals and not release pollutants. v) Environmental stability. The performance of paper strongly depends on the relative humidity (RH). Due to the hygroscopicity, high RH will reduce its dimensional stability and strength. vi) Cost. The cost of papermaking and electronics fabrication, both, have a great influence on the scale‐up potential of paper electronics.

**Figure 4:**
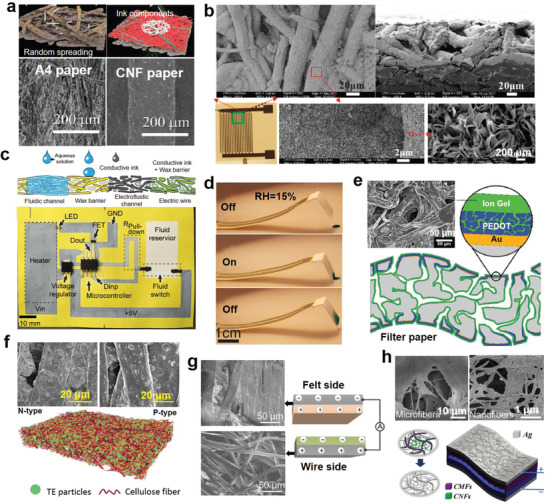
Examples of using the porous structure of papers to realize new functions and improve device performance. a) Controlling the printing resolution by adjusting the paper's capillarity. Reproduced with permission.^[^ ^62^ ^]^ Copyright 2016, The Royal Society of Chemistry. b) Optical and SEM images of the paper‐based capacitor fabricated by copper ELD. Reproduced with permission.^[^ ^35^ ^]^ Copyright 2018, Wiley‐VCH. c) Integrating electronics and microfluidics on paper. Reproduced with permission.^[^ ^36^ ^]^ Copyright 2016, Wiley‐VCH. d) Hygroexpansive electrothermal paper actuator activated by the change of moisture content. Reproduced with permission.^[^ ^37^ ^]^ Copyright 2016, Wiley‐VCH. e) Monolithic flexible SCs integrated into single sheets of paper. Reproduced with permission.^[^ ^67^ ^]^ Copyright 2017, Wiley‐VCH. f) Porous paper enables a thick deposition layer of TE particles without blocking the pores. Reproduced with permission.^[^ ^59^ ^]^ Copyright 2019, American Chemical Society. g) TENG based on heterostructured air‐laid paper. Reproduced with permission.^[^ ^38^ ^]^ Copyright 2019, Wiley‐VCH. h) Hierarchically nanostructured cellulose fiber‐based TENG. Reproduced with permission.^[^ ^75^ ^]^ Copyright 2018, Wiley‐VCH.

Among various commercially available papers, printing paper, copy paper, filter paper, and chromatography paper are ideal substrates for manufacturing low‐cost electronics. The porous structure of paper derives many unique properties, which can help paper‐based electronics realize new functions and improve device performance (**Figure** **4**). For example, the printing resolution can be improved by adjusting the capillarity of paper.^[^ ^62^ ^]^ As shown in Figure 4a, by inkjet printing a layer of cellulose nanofibers (CNF) on A4 paper to control the capillary force, high‐resolution print patterns can be obtained. On the other hand, the ink penetration induced by capillarity is conducive to the adsorption of active substances and can promote the electroless deposition (ELD) and metallization of paper. Using ELD, various functional paper‐based devices including electrodes,^[^ ^64^ ^]^ SCs,^[^ ^35^ ^]^ and radio frequency identification (RFID) tags^[^ ^65^ ^]^ have been achieved. Figure 4b demonstrates a high‐performance all‐solid‐state SC developed by Yang's group.^[^ ^35^ ^]^ Thanks to the porous cellulose fibers, the engraved Cu_x_O nanostructures evenly covered every metalized fiber in the catalyst‐activated area. The capillarity can also be used to integrate electronic and microfluidic components on paper.^[^ ^36^ ^]^ Figure 4c exhibits an integrated paper‐based electrofluidic device fabricated by wax printing and poly(3,4‐ethylenedioxythiophene):polystyrene sulfonate (PEDOT:PSS) ink deposition. In addition, paper‐based hygroexpansive electrothermal actuators^[^ ^37^ ^]^ and humidity sensors^[^ ^66^ ^]^ have been designed based on the hygroscopicity of paper. As shown in Figure 4d, the moisture content change in the porous conductive path can activate the paper actuator.

In addition to absorbing liquid, the porous structure of paper also helps the storage and bonding of solid active materials. For instance, Liu et al.^[^ ^67^ ^]^ built a monolithic flexible SC based on a single sheet of filter paper (Figure 4e). PEDOT was deposited on both sides of the paper as electrodes. The paper not only functions as the separator but also facilitates solid‐state electrolyte storage. Owing to its large storage capacity, the fabricated SC demonstrates high volumetric capacitance and energy density. Similarly, the pores of paper can promote the insertion and adhesion of thermoelectric materials^[^ ^68^ ^]^ and conductive composites^[^ ^69^ ^]^ to produce high‐performance thermoelectric generators^[^ ^59^ ^]^ and conductive tracks.^[^ ^70^ ^]^ Figure 4f presents the thick Bi_2_Te_3_ alloy deposition achieved on paper without blocking the pores. Moreover, the rough paper is good for making high‐performance triboelectric nanogenerator (TENG) ^[^ ^71^ ^]^ and batteries.^[^ ^72^ ^]^ Due to the unique heterogeneous structure of the air‐laid paper, Yang et al.^[^ ^38^ ^]^ utilized its two sides as the friction pair of a wearable TENG (Figure 4g). Their rough surfaces and distinct triboelectric properties greatly strengthen the triboelectric effect, leading to an exceptional output performance. Inspired by the lemon battery, Wang et al.^[^ ^72^ ^]^ created fully degradable fruit‐based batteries with high areal capacity (2.9 µAh cm^−2^ at 40 µA cm^−2^) and energy density (around 4.0 µW h cm^−2^ at 56 µW cm^−2^ power density) on cellulose paper. The rough and porous paper substrate provides a large contact surface area and promotes electrolyte penetration, contributing to its excellent electrochemical performance.

Apart from commercially available papers, customized papers with desired properties can be obtained via modified papermaking techniques. Nanocellulose paper (NCP) composed of CNF is a representative customized paper. Compared with micro cellulose paper, NCP has better softness, light transmittance, and barrier function. Incorporating different functional ingredients to NCP can bring various desirable properties. For instance, transparent conductive NCP with enhanced mechanical properties and chemical corrosion stability has been developed by mixing oxidized nanocrystalline cellulose, chitosan, and silver nanowires (Ag NWs).^[^ ^73^ ^]^ Similarly, Oh et al.^[^ ^74^ ^]^ designed highly conductive ferroelectric NCPs with an improved electron‐donating tendency by mixing Ag NWs, BaTiO_3_ nanoparticles, and bacterial cellulose for high‐performance TENGs. Figure 4h shows a hierarchically nanostructured cellulose fiber‐based TENG reported by Wang's group.^[^ ^75^ ^]^ CNF was coated on the cellulose microfibers (CMFs) to optimize its pore structures, which not only enhanced the electrification effect but also enabled particulate matter removal.

#### 2.1.2 Fabrics

**Figure 5:**
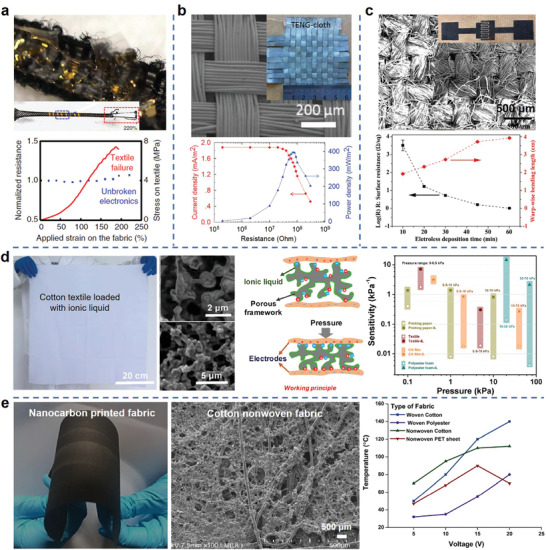
Examples of using the porous structure of fabrics to improve device performance include stretchability, triboelectric output performance, conductivity, sensitivity, and heat dissipation rate. a) The stretchable fabric offers strain limiting support to keep the device's integrity until 220% strain. Reproduced with permission.^[^ ^39^ ^]^ Copyright 2014, Springer Nature. b) A whole‐textile TENG by weaving Ni‐coated polyester fabric electrodes with high power density. Reproduced with permission.^[^ ^40^ ^]^ Copyright 2015, Wiley‐VCH. c) A highly conductive interdigital electrode fabricated on a cotton fabric via Ni ELD. Reproduced with permission.^[^ ^83^ ^]^ Copyright 2019, Wiley‐VCH. d) Ionic liquid activated wearable sensors based on cotton fabric with enhanced sensitivity. Reproduced with permission.^[^ ^41^ ^]^ Copyright 2019, Elsevier. e) Highly porous heating fabrics with exceptional heat dissipation properties. Reproduced with permission.^[^ ^87^ ^]^ Copyright 2018, Elsevier.

Fabrics are porous, highly deformable 3D fiber assemblies. Unlike the random connection of the paper cellulose fibers, the fabric fibers are woven or knitted to form a tightly interlocking structure as shown in **Figure** **5**. This unique fiber assembly endows the fabric with stronger mechanical strength, higher flexibility, bigger porosity, better elasticity, and recovery ability. Fabrics have been regarded as the second skin of humans for thousands of years. The first application of fabric‐based electronics dates back to the use of light‐emitting headbands in the ballet La Farandole in 1883.^[^ ^76^ ^]^ In recent years, fabrics have attracted much interest for the fabrication of wearable electronics, because of their irreplaceable advantages such as light weight, softness, comfort, warmth retention, breathability, durability, and washability. Fabric‐based electronics can be easily integrated into garments and work in an imperceivable way.^[^ ^26b^ ^]^ Besides, combined with mature fabric mass production methods, low‐cost fabric‐based electronics can be realized for daily healthcare monitoring, physical and chemical sensing, energy harvest, and storage.

There are more than 200 types of commercially available fabrics. Based on the manufacturing techniques, they are divided into woven, knitted, braided, knotted, etc.^[^ ^5^ ^]^ According to the raw materials, fabrics can be classified into natural, synthetic, and blended. Among them, cotton, silk, polyester, and elastic fabrics^[^ ^77^ ^]^ are commonly used in wearable electronics. The basic properties of the fabric, including porosity, breathability, strength, softness, elasticity, and absorbency, are mainly determined by the type of yarn and the fabric production method. Combined with well‐established fabric processing technology, electronic devices can be readily constructed on fabric. For example, miniaturized ready‐made electronics can be integrated into fabric by stitching or embroidering. The fiber‐shaped electronics are easily weaved,^[^ ^78^ ^]^ knitted,^[^ ^79^ ^]^ and sewed^[^ ^80^ ^]^ in fabric. Here, we will mainly discuss the manufacture of electronic devices directly on fabrics, and some representative cases are displayed in Figure 5.

The advantages of fabric substrates and the role of their porous structures in improving device performance are summarized as follows: i) Fabric substrate can provide strong mechanical support for electronic devices because the robust interlocking fiber structure brings outstanding stretchability and superior fatigue/crack resistance. Figure 5a presents a skin‐mounted physiological monitoring device fabricated on a stretchable fabric, where the fabric offers strain limiting support to keep the device integrity. The system did not start to fail until 220% strain.^[^ ^39^ ^]^ ii) Similar to paper, the porous and rough fabric is conducive to the construction of high‐performance TENG.^[^ ^81^ ^]^ Wang's group designed a whole‐textile TENG by weaving Ni‐coated polyester fabric electrodes as shown in Figure 5b. The microscale roughness of the polyester cloth not only promotes the friction contact area but also enhances the adhesion of the conductive Ni film, thereby boosting the electrical output performance.^[^ ^40^ ^]^ The peak power density can reach a maximum of 393.7 mW m^−2^ when the external resistance is about 70 MΩ. iii) The capillarity and hydrophilicity of fabric facilitate the absorption of functional inks and promote the binding of active materials. For example, highly conductive fabrics have been fabricated by ELD because the fabric can enhance the absorption and penetration of precursor catalytic ions, and help the subsequent ELD to generate strongly bonded metal nanoparticles, thus improving conductivity.^[^ ^82^ ^]^ Figure 5c illustrates an interdigital electrode fabricated on a cotton fabric via Ni ELD, which can see the tight adhesion between Ni and cotton fibers.^[^ ^83^ ^]^ When the electroless plating time is 60 min, the surface resistance of as‐made conductive fibers can be as low as 1.02 Ω sq^−1^. iv) The pores in the fabric provide multi‐functional material exchange channels. They can accelerate the electrolyte ion diffusion and increase the electrolyte/active material interface,^[^ ^84^ ^]^ therefore improving the power density of SC^[^ ^85^ ^]^ and electrochemical performances of fabric‐based electrodes.^[^ ^86^ ^]^ In addition, the porous structure can provide enough gas effusion channels to achieve highly sensitive gas sensors.^[^ ^42^ ^]^ For instance, by immersing the cotton fabric in ionic liquid, Yang et al.^[^ ^41^ ^]^ developed wearable sensors with fast responses (tens of milliseconds) and high sensitivity (up to 10 kPa^−1^). The micromorphology of the ionic liquid‐coated porous structure is shown in Figure 5d. v) The pore structures can function as energy dissipation channels. To fabricate wearable heating fabrics, Arbab et al.^[^ ^87^ ^]^ printed multiwall carbon nanotubes (MWCNTs) on different fabrics. The obtained porous nonwoven cotton sample with the highest heat dissipation rate is demonstrated in Figure 5e. Besides, cotton fabrics coated with reduced graphene oxide (RGO)/Ti_3_C_2_T_x_ MXenes were used to absorb electromagnetic waves and showed excellent electromagnetic interference shielding efficiency (36.62 dB under X‐brand).^[^ ^88^ ^]^

#### 2.1.3 Electrospun Nanofibers

The electrospun nanofibers are composed of highly porous nonwoven nanofiber networks. It combines many advantages of paper and fabric, including light weight, transparency, biocompatibility, breathability, flexibility, stretchability, comfort, washability, and stitchability.^[^ ^9^ ^]^ Because electrospinning can accurately control the nanofiber structure (i.e., diameter, morphology, and orientation) and arrange materials at the molecular level, electrospun nanofibers possess enhanced surface area, controllable porosity, extremely long nanofibers, and customized micro/nanostructures. These remarkable features make them attractive materials for flexible sensors and energy devices.^[^ ^27^ ^]^ In addition, electrospun nanofibers made of biocompatible polymers that can be directly applied on the skin or in vivo have been widely used in epidermal electronics and implantable electronics.

Since the invention of electrospinning in 1943, over 100 different types of organic polymers have been utilized to produce continuous nanofibers.^[^ ^89^ ^]^ The polymer precursors can be synthetic polymers such as PU, polyvinylidene fluoride (PVDF), polyvinyl alcohol (PVA), and polyacrylonitrile (PAN), or natural polymers such as collagen, silk, and chitosan.^[^ ^9^ ^]^ During electrospinning, a high‐voltage electric field is applied to the polymer precursor solutions to eject electrically charged jets from the spinneret, then the polymer jets are stretched and accelerated toward the collector plate. As the solvent evaporates, nanofibers are continuously formed and accumulated to form a non‐woven mat.^[^ ^27^ ^]^ Its porosity, thickness, and fiber structures can be tailored by changing the processing parameters. Critical solution parameters include the type of polymer, concentration, viscosity, surface tension, and conductivity. Operational parameters such as applied voltage, flow rate, and tip‐to‐collector distance also play vital roles in fiber morphology. In addition, environmental parameters such as temperature and humidity can affect the crystallization and phase separation process of the polymer, thus affecting the fiber morphology.^[^ ^89^ ^]^ Functional nanofibers can be easily obtained by doping active nanomaterials in the precursor solution or performing surface modification after electrospinning.^[^ ^90^ ^]^ Representative functional nanomaterials include metals (e.g., Au, Ag), metal oxides (e.g., TiO_2_, WO_3_), carbon materials (e.g., graphene, CNTs), and their composites.^[^ ^91^ ^]^

**Figure 6:**
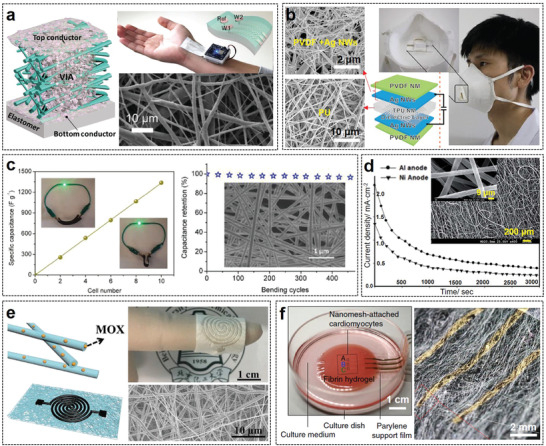
Examples of using electrospun nanofibers to realize various high‐performance flexible electronics. a) Two‐layered e‐textile patches. Reproduced with permission.^[^ ^16^ ^]^ Copyright 2018, Wiley‐VCH. b) Breathable pressure sensor based on electrospun PVDF. Reproduced with permission.^[^ ^14^ ^]^ Copyright 2017, Elsevier. c) Electrospun PANI‐based flexible SC with high specific capacitance and superior capacitance retention capability. Reproduced with permission.^[^ ^43^ ^]^ Copyright 2019, Wiley‐VCH. d) Electrospun PVDF nanofiber substrate with hybrid randomly oriented and aligned morphologies are used to prepare implantable fuel cells to achieve enhanced species material transport. Chronoamperometry curves were measured at 0.3 and 0.15 V for the single‐compartment paper‐based H_2_O_2_ fuel cells with different anodes. Reproduced with permission.^[^ ^95^ ^]^ Copyright 2017, Royal Society of Chemistry. e) On‐demand anti‐infection therapy device based on electrospun nanofibers. Reproduced with permission.^[^ ^44^ ^]^ Copyright 2019, Wiley‐VCH. f) Electrospun PU‐based ultrasoft electronics to monitor dynamically pulsing cardiomyocytes. Reproduced with permission.^[^ ^45^ ^]^ Copyright 2019, Springer Nature.

Similar to paper and fabric, the porous structure of electrospun nanofibers is also conducive to improving device performance, such as sensitivity,^[^ ^92^ ^]^ energy density, conductivity, and stretchability, as shown in **Figure** **6**.^[^ ^93^ ^]^ By controlling the penetration of conductive ink, Chung's group^[^ ^16^ ^]^ designed two‐layered e‐textile patches based on porous electrospun nanofibers for electromyography and electroencephalography sensing. As shown in Figure 6a, sufficient penetration depth enables vertically interconnected accesses (VIA). Therefore, the sensing electrodes and the conducting traces can be separated on the opposite sides of the substrate, providing a more comfortable and safe wearing experience. Utilizing the excellent air permeability of electrospun substrates, Yang et al.^[^ ^14^ ^]^ created a breathable pressure sensor with high sensitivity, as exhibited in Figure 6b. The sensor consists of a layer‐by‐layer structure of electrospun PVDF for substrates, Ag NWs for electrodes, and electrospun PU for the dielectric layer. The PU dielectric layer provides a large deformation space, making the sensor sensitive to pressure. The porous PVDF substrates can accelerate the airflow velocity in the PU layer, thus further improving the response speed. In addition, flexible SCs with high specific capacitance and superior capacitance retention capability (Figure 6c), have been developed based on electrospun polyaniline (PANI) nanofibers.^[^ ^43^ ^]^ The continuous nanofiber networks not only endow the device with great stress tolerance but also facilitate fast charge transfer and increase ion storage capacity.

Electrospun nanofibers made of biocompatible/biodegradable polymers are ideal substrates for flexible electronics that need direct contact with cells, tissues, or organs because their pore interconnection can mimic the texture and compositions of diversified extracellular matrix and interact with cells.^[^ ^94^ ^]^ For instance, based on biocompatible PVDF nanofibers, Asadnia et al.^[^ ^95^ ^]^ built an implantable fuel cell with excellent mechanical properties and chemical stability. The elastic modulus and hardness of a single PVDF nanofiber are 2.2 and 0.1 GPa, respectively. By optimizing electrospinning parameters, PVDF nanofibers with hybrid randomly oriented and aligned morphologies were obtained as shown in Figure 6d. These customized nanofibers are especially useful for improving the fuel cell performance by enhancing species transport on the surface and reducing concentration loss. The recorded chronoamperometry curves of the paper‐based H_2_O_2_ fuel cells with different anodes indicate the good durability and chemical stability of the cell performance. Biocompatible electrospun nanofibers can also work as drug carriers by adding drugs to the precursor polymer solution. Figure 6e illustrates an on‐demand antiinfection therapy device produced by printing conductive patterns on moxifloxacin hydrochloride (MOX)‐loaded electrospun nanofibers.^[^ ^44^ ^]^ The high porosity (71.2%) and deformability of the substrate bring excellent air and liquid permeability and good compatibility with dynamic tissues. Moreover, Lee et al.^[^ ^45^ ^]^ developed an ultrasoft electrospun PU nanomesh device that can monitor the dynamically pulsing cardiomyocytes. Owing to the porous nanomesh (Figure 6f), nutrients and wastes can freely cross the cell/nanomesh interface and penetrate the culture medium. Because of its extraordinary flexibility (Young's modulus of 274 kPa) and stretchability (the resistance of the nanomesh electrodes is 211.6  ± 63.4 Ω at tensile strains of 20%), the contraction and relaxation of cardiomyocytes are completely unrestricted by the attached device.

#### 2.1.4 Natural Porous Materials

**Figure 7:**
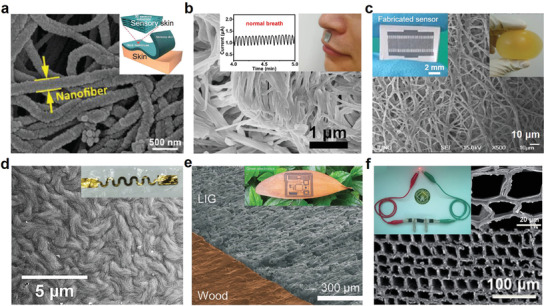
Representative natural porous substrates for biodegradable and green electronics. a) Cowskin‐based breathable, humidity‐ultrastable sensory skin. Reproduced with permission.^[^ ^46^ ^]^ Copyright 2019, Royal Society of Chemistry. b) Collagen leather‐based humidity sensor. Reproduced with permission.^[^ ^98^ ^]^ Copyright 2019, American Chemical Society. c) Inner eggshell membrane‐based humidity sensor. Reproduced with permission.^[^ ^47^ ^]^ Copyright 2019, Springer Nature. d) Ovine collagen film for flexible implantable electronics. Reproduced with permission.^[^ ^48^ ^]^ Copyright 2015, Wiley‐VCH. e) Laser‐induced graphene on woods and leaves for green electronics. Reproduced with permission.^[^ ^99^ ^]^ Copyright 2019, Wiley‐VCH. f) Wood‐based all‐solid‐state flexible SCs. Reproduced with permission.^[^ ^100^ ^]^ Copyright 2015, Royal Society of Chemistry.

Natural materials such as woods, leaves, and animal skins have inherently hierarchical pore‐rich 3D structures, such as well‐organized honeycomb and densely packed cellular units as shown in **Figure** **7**. The architecture design in nature is extremely efficient, light, and strong, with exceptional topographic features and in situ‐tailorability of chemical composition.^[^ ^10^ ^]^ Compared with manmade porous substrates, these natural porous materials possess many unparalleled advantages, including renewability, low cost, biodegradability, biocompatibility, self‐cleaning properties, antimicrobial activity, and other unique functionalities. Their superior properties stem from their unique hierarchical structures which have been finely evolved over millions of years. For example, natural pore structures usually exhibit remarkable transport performance because they are meticulously designed by nature for nutrient transport, waste metabolism, heat dissipation, and breathability. In addition to the unique structural features, natural porous materials contain many functional groups, such as hydroxyl, carboxyl, amino, and amine groups, which enable them to have superb functions (e.g., recognition, selective adsorption, and sensing) beyond the limits of synthetic materials.^[^ ^96^ ^]^ In recent years, “green” or “sustainable” electronics has become very popular which promises to integrate electronics into living tissues by human and environmentally friendly technologies to achieve multitasks including real‐time biochemical monitoring, diagnostic, and drug delivery.^[^ ^97^ ^]^ To realize this goal, lots of natural materials including plants, active organisms, and biological molecules have been explored as electrodes, active materials, substrates for sensors, transistors, SCs, batteries, and electronic displays. Next, we will list some examples of using natural porous substrates to help electronic devices achieve specific functions.

According to the material source, the natural porous substrates are categorized into animal‐based and plant‐based. Due to excellent skin affinity, animal‐based porous substrates are often used in on‐skin electronics. For example, cowskin has similar constituents and hierarchical structure to human skin, showing high skin conformability, mechanical robustness, and humidity stability. Using a treated cowskin, Ke et al.^[^ ^46^ ^]^ constructed a highly breathable and humidity‐stable sensory skin device. The basic building blocks of the cowskin are collagen nanofibers with a diameter of 50–200 nm as shown in Figure 7a. Owing to the interwoven fibrous structure, the sensory skin displayed remarkable breathability, exceptional water‐vapor permeability (3714 g m^−2^ d^−1^), and a wide sensory range from weak physiological signals to high strain movements. After physical and chemical treatment such as depilation and tanning, animal skin will denature into nonperishable leather maintaining a tightly woven 3D structure of natural protein fibers. Figure 7b demonstrates the highly porous hierarchical structure of collagen leather. Taking advantage of its high porosity, hygroscopicity, and biocompatibility, Xie et al.^[^ ^98^ ^]^ fabricated a highly sensitive humidity sensor for real‐time respiration monitoring. Similarly, natural inner eggshell membrane (IESM) has been utilized as the substrate for a humidity sensor.^[^ ^47^ ^]^ The IESM is about 19 µm thick and exhibits porous multilayer crosslinked fiber structures as shown in Figure 7c. The porous IESM not only works as the ultra‐soft substrate for the sensor but also acts as the active sensing layer, showing excellent moisture sensitivity (response time ≈1.99 s). In addition to on‐skin applications, animal‐derived purified collagen can be safely implanted. Moreno and co‐authors^[^ ^48^ ^]^ successfully demonstrated diverse implantable electronics (i.e., strain gauge, wireless antenna, heater, and temperature sensor) on collagen films prepared from ovine collagen solution. Figure 7d presents the uniform porous and filamentous microstructure of the transparent collagen films. This fibrous microstructure makes the collagen film insoluble in water and saline solutions, which is essential for maintaining its integrity after implantation in vivo.

Plant‐based substrates such as woods and leaves are “greener” earth‐abundant materials. Using programmable laser irradiation, Kim's group^[^ ^99^ ^]^ designed diverse graphene‐based green electronics including electrical interconnects, temperature sensors, and pseudocapacitors on woods and leaves. Figure 7e illustrates the porous structure of laser‐induced graphene on wood, which is generated by the decomposition of organic materials, rapid liberation of gaseous products, and pre‐existing microstructures. The highly conductive porous graphene provides a large specific surface area and enhances the charge carrier mobility (electrical conductivity of 42 Ω sq^−1^), thus improving the device's performance (areal capacitance 53.6 mF cm^−2^ at 1 mA cm^−2^). It is well‐known that woods are anisotropic fibrous materials and have “well‐aligned” tubular structures along the wood growth direction which facilitate the water, salts, and other nutrients transportation from roots to leaves. Inspired by this natural transport property, delignified nanowood biofilms were exploited as sweat and heat guiding layers for green on‐skin electronics for biosensing and targeting drug delivery.^[^ ^21^ ^]^ In addition, the pore structures of woods are also ideal reservoirs and channels for electrolyte absorption. Using a natural porous wood slice, Lv et al.^[^ ^100^ ^]^ developed a high‐performance all‐solid‐state flexible SC. The 3D honeycomb porous framework of wood is shown in Figure 7f, which greatly improves the electrochemical performance (capacitance of 0.61 F cm^−2^) of the SC by providing high specific surface areas to fully utilize active materials and offering transportation corridors to promote electrolyte transport.

### 2.2 Non‐Fiber‐Based Substrate Materials

Due to their exceptional stretchability and deformation recovery capability, porous organic elastomers such as PDMS and PU are widely applied in stretchable electronics. Different porous structures can be introduced into the elastomers by diverse pore‐forming technologies such as direct templating, emulsion templating, gas foaming, phase separation, and 3D printing.^[^ ^101^ ^]^ Generally, the introduction of porous structures can greatly improve the performance of elastomers by increasing their surface area, facilitating the incorporation of functional materials, and promoting their chemical activity. Currently, a wide range of electronic devices including flexible conductors, stretchable sensors, batteries, and SCs have been successfully fabricated on porous elastomers.

#### 2.2.1 Porous PDMS

**Figure 8:**
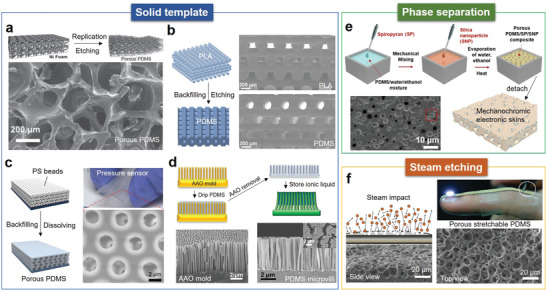
Porous PDMS substrates manufactured by different methods are used in compressible/stretchable electronics. a) Highly porous PDMS with continuous macropores fabricated with Ni foam template. Reproduced with permission.^[^ ^102^ ^]^ Copyright 2014, Wiley‐VCH. b) Stretchable PDMS conductors produced with 3D printed PLA scaffolds. Reproduced with permission.^[^ ^103^ ^]^ Copyright 2015, American Chemical Society. c) Highly sensitive PDMS pressure sensor with multilayered pores of different sizes using PS bead template. Reproduced with permission.^[^ ^17^ ^]^ Copyright 2016, Wiley‐VCH. d) Porous PDMS with biomimetic microvilli structures manufactured with the AAO mold for self‐healing electronics. Reproduced with permission.^[^ ^49^ ^]^ Copyright 2019, American Chemical Society. e) Porous PDMS‐based mechanochromic electronic skin fabricated by phase separation. Reproduced with permission.^[^ ^106^ ^]^ Copyright 2019, Wiley‐VCH. f) Porous PDMS produced by steam etching for stretchable circuits. Reproduced with permission.^[^ ^50^ ^]^ Copyright 2012, Springer Nature.

PDMS has become the most popular silicone‐based organic elastomer since the first batch of silicone was synthesized in the 1950s.^[^ ^101^ ^]^ Polymer chains in PDMS are chemically crosslinked by strong covalent bonds.^[^ ^11^ ^]^ The Si–O–Si backbone renders it many intriguing properties including low bulk density, stable chemical properties, good thermal and electrical stability, mechanical robustness, high flexibility, and biocompatibility. Optically, PDMS exhibits high transmittance and low absorption under ultraviolet (UV) irradiation. In terms of processability, PDMS has superb moldability with fine contour accuracy of less than 10 nm. Its mechanical property can be easily adjusted by changing the amounts of curing agents or the crosslinking temperature.^[^ ^11^ ^]^ In addition to these intrinsic properties, the introduction of porous structures can further enhance deformability, breathability, surface activity, and facilitate PDMS functionalization. Porous PDMS can be functionalized by adding functional materials during pore formation or surface modification after pore formation. The pore structure (e.g., morphology, porosity, size, interconnectivity, and distribution) can be changed by different pore‐forming techniques utilizing various templates or porogen materials. Owing to its controllable physical and chemical properties, outstanding deformability, and good moldability, porous PDMS has become an attractive substrate material for stretchable electronics. **Figure** **8** gives some examples of using porous PDMS substrates manufactured by different methods to achieve electronic devices with various functions.

In the solid template method, the template is first immersed in the precursor PDMS solution, and the pore structure can be formed by removing the template after the PDMS is cured. Soluble metals and polymers are typical solid templates. For example, Chen et al.^[^ ^102^ ^]^ fabricated a porous PDMS with continuous macropores through a Ni foam template, as shown in Figure 8a. The obtained porous PDMS was functionalized by dip‐coating with hybrid CNT/RGO to realize a highly stretchable conductor. The pore structure not only improves conductivity by providing a 3D template for building nanomaterial networks but also increases working stability by enhancing its load‐bearing capacity. The porous PDMS conductor exhibits high electrical conductivity with a low nanofiller loading (27 S m^−1^ with 2 wt% CNTs/graphene) and its conductivity remains constant even as the strain increases to 50%. Li's group^[^ ^103^ ^]^ manufactured high‐performance stretchable PDMS conductors with the 3D printed polylactic acid (PLA) scaffold templates (Figure 8b). The pore architecture of PDMS is determined by the structure of the PLA scaffold. Changing the pore structures can alter the stress distribution in the PDMS conductor and bring about distinct conductivity retention capabilities. Apart from stretchable conductors, porous PDMS is also commonly employed in flexible sensors.^[^ ^104^ ^]^ By imitating the natural multilayered porous structures in mushrooms, Kang et al.^[^ ^17^ ^]^ designed a PDMS pressure sensor with multilayered pores of different sizes using the polystyrene (PS) bead template (Figure 8c). Compared to the unstructured pressure sensor, the hierarchical porous structure considerably improved the sensor's sensitivity (0.63 kPa^−1^, low‐pressure detection of 2.42 Pa) by increasing the elasticity and effective dielectric constant of the dielectric layer. Similarly, a PDMS strain sensor with tunable stretchability was fabricated with a PS sphere array template.^[^ ^105^ ^]^ Compared with the bulk PDMS, the introduction of the pore structure increases the stretchability by 210%, and this enhancement can be controlled by the pore size and the porosity of the PDMS. In addition to super stretchability, the self‐healing property is also critical to the performance of stretchable PDMS electronics. Inspired by the abrasion‐resistant structures of tear films on animal corneas, an ultradurable self‐healing strain sensor has been created by Miao et al.^[^ ^49^ ^]^ with an aluminum oxide (AAO) template. Figure 8d displays the biomimetic microvilli structures of the porous PDMS, which was used to store ionic liquids. Owing to the capillary‐force‐induced self‐healing capability of the porous PDMS, the strain sensor exhibited high durability of over 22 500 loading–unloading cycles.

Phase separation techniques using liquid porogen such as hydrophilic solvents are also able to produce porous PDMS. By employing hydrophilic cosolvents composed of water and ethanol, Park et al.^[^ ^106^ ^]^ developed a functional porous PDMS with a hierarchical nanoparticle‐in‐micropore structure for mechanochromic e‐skins. The porous structure is shown in Figure 8e, which greatly enhanced the device's sensitivity (mechanochromic color change at low strain/stress values (tensile strain: 50%; normal force: 1 n)) and stretchability (reversible mechanochromism under a strain of 250%). In addition, steam etching applying pressurized steam to impact the surface of uncured PDMS is effective to produce hierarchical porous PDMS as illustrated in Figure 8f.^[^ ^50^ ^]^ Combining steam etching and metal deposition, solderable and electroplatable PDMS‐based flexible circuits have been successfully generated. Due to the highly stretchable porous PDMS, the resulting circuits exhibited superb conductivity retention capability which survived over 20 000 stretching cycles and could be bent beyond 150° without significant degradation of conductivity.

#### 2.2.2 Porous PU

PU is composed of hard blocks (urethane groups) and soft blocks (polyols) with chemical and physical crosslinking. In its networks, urethanes are chemically crosslinked with isocyanate groups through allophanate bonds and physically crosslinked with each other through hydrogen bonds.^[^ ^11^ ^]^ The properties of PU can be controlled by adjusting the types and ratios of polyols and polyisocyanates. PU is one of the most broadly used thermoplastic polymers by virtue of its outstanding processability, biocompatibility, high tensile strength, and chemical resistance.^[^ ^12^ ^]^ The introduction of pore structures brings many additional advantages to PU substrates, such as lightweight, breathability, excellent flexibility, and high strength‐to‐weight ratio. There are a lot of well‐developed technologies to produce porous PU, such as gas foaming, thermally induced phase separation, emulsion freeze‐drying, solvent casting/particulate leaching, and melt molding.^[^ ^107^ ^]^ Depending on the composition and synthesis method, the properties of porous PU vary greatly, from hydrophobic to hydrophilic, from thermoplastic to thermosetting, from stable to degradable. The pore structures can be tuned by changing the porogen agent, solvents, and thermal processing parameters. Porous PU can be readily functionalized by adding nanofillers (e.g., graphene, CNT, Ag NWs, nanoclay, nanosilica) to generate compressible/stretchable sensors.

**Figure 9:**
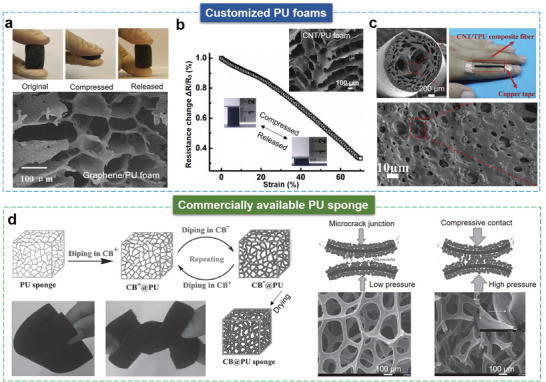
Customized PU foams and commercially available PU sponge for compressible/stretchable sensors. a) Highly compressible graphene/PU foams with an interconnected cell structure fabricated by the thermal‐induced phase separation for the piezoresistive sensor. Reproduced with permission.^[^ ^13^ ^]^ Copyright 2017, The Royal Society of Chemistry. b) The CNT/PU foam with herringbone structures produced by the directional ice‐template freezing shows a linear piezoresistive property. Reproduced with permission.^[^ ^108^ ^]^ Copyright 2017, Elsevier. c) Highly stretchable porous MWCNTs/PU fiber fabricated by the wet‐spun method for human motion monitoring. Reproduced with permission.^[^ ^51^ ^]^ Copyright 2018, Elsevier. d) A versatile pressure sensor based on CB@PU sponge for HMI. Reproduced with permission.^[^ ^34^ ^]^ Copyright 2016, Wiley‐VCH.

As shown in **Figure** **9**, customized PU foams with diverse pore structures can be produced by different foaming techniques. Before the foaming process, conductive nanomaterials are often added to the PU precursor solution to achieve piezoresistive PU foam. Different porous architectures can result in distinct deformability and piezoresistive properties. For example, using the thermal‐induced phase separation technique, Liu et al.^[^ ^13^ ^]^ fabricated lightweight conductive graphene/PU foams with an interconnected cell structure as shown in Figure 9a. The density and porosity of the foams were about 0.11 g cm^−3^ and 90%. Owing to the unique porous structure, the PU foam exhibits special positive piezoresistive behaviors, good compressibility, and stable sensing at a strain of up to 90%. Applying the directional ice‐template freezing, Wei et al.^[^ ^108^ ^]^ developed a CNT/PU foam with herringbone structure, showing a linear piezoresistive property as illustrated in Figure 9b. The novel herringbone porous structure greatly improves the foam's working stability even under large deformation, because the pore structure is supported by the skeleton morphology and connects one by one through the cell strut. Figure 9c presents a highly stretchable porous MWCNTs/PU fiber fabricated by the wet‐spun method for human motion monitoring.^[^ ^51^ ^]^ Owing to its high porosity, elasticity, and recoverability, the sensor displays an ultra‐wide strain range (320%), high sensitivity (gauge factor of 22.2 within 160% strain and 97.1 for a strain of 160–320%), good reproducibility, and durability (9700 stretching‐releasing cycles at 100% strain).

Commercially available PU sponges are usually dip‐coated with conductive materials (e.g., RGO^[^ ^109^ ^]^ and Ag NWs)^[^ ^110^ ^]^ to make piezoresistive PU composites. Other nanomaterials such as aramid nanofibers ^[^ ^111^ ^]^ and CNF^[^ ^112^ ^]^ can be added to improve the adhesion of conductive layers and enhance the mechanical properties of composites. The functionalized PU sponges have attracted great attention for the production of compressible pressure sensors and stretchable gas sensors. Because PU has a large number of hydrophilic groups, which is conducive to the absorption and chemical bonding of nanofillers. In addition, the porous framework provides a large sensing area and reduces the stress concentration on the conductive nanofiller, thereby improving the sensitivity and reproducibility of the sensor. For instance, inspired by the spider sensory system, Lu's group^[^ ^34^ ^]^ reported a versatile carbon black (CB) @PU‐based pressure sensor for the human–machine interface (HMI). Thanks to the microcrack‐designed porous structure, the sensor can detect both tiny (91 Pa pressure, 0.2% strain) and large motions (16.4 kPa pressure, 60% strain), exhibiting a fast response time (<20 ms) and good reproducibility (over 50 000 cycles). As shown in Figure 9d, the detection mechanisms of tiny and large motions are based on the microcrack junction sensing and compressive contact of CB@PU conductive backbones, respectively. Besides, owing to the medical‐grade biocompatibility and excellent breathability, semi‐permeable PU films are ideal substrates for adhesive on‐skin sensors for long‐term monitoring of temperature,^[^ ^113^ ^]^ pulse, and body motion.^[^ ^114^ ^]^ Their nanoscale pore structures are permeable to water vapor and oxygen but not water and bacteria. Moreover, their waterproof performance can protect the functional layer from short circuits.

## 3 Functionalization Methods of Flexible Porous Substrate

Most of the aforementioned porous substrates are electrically nonconductive materials. To improve their conductivity and build electronic components on them, conductive materials need to be coated on or penetrated porous substrates. To date, a variety of functionalization methods have been developed to integrate different types of conductive materials into porous substrates. For example, metal nanoparticles (e.g., Ag, Ni, Cu, Au, Cr) and carbon‐based materials (e.g., CNTs, graphene, CB) dispersed in inks or paste can be printed or coated on porous substrates. Graphene can be obtained on porous substrates by in situ laser graphitization. Conductive polymers (e.g., PEDOT:PSS, PANI, polypyrrole [PPy]) can be coated on porous substrates by electrodeposition or in situ polymerization. According to different manufacturing processes, functionalization methods are grouped into physical methods and chemical methods.

### 3.1 Physical Methods

**Figure 10:**
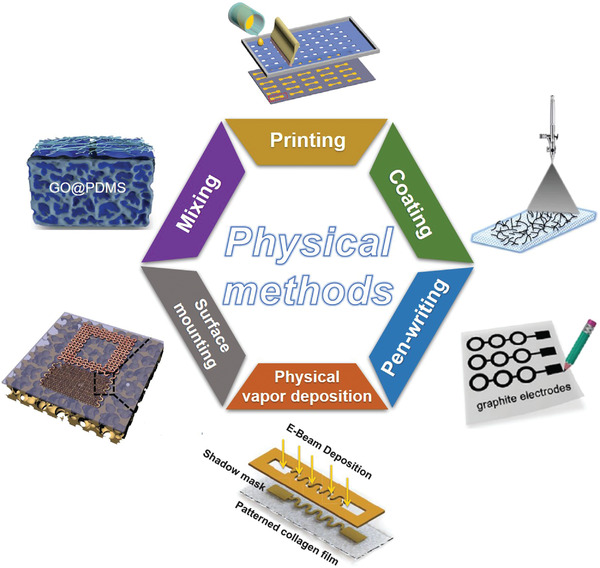
Physical methods for the functionalization of flexible porous substrates. “Printing”: Reproduced with permission.^[^ ^115^ ^]^ Copyright 2018, Royal Society of Chemistry. “Coating”: Reproduced with permission.^[^ ^116^ ^]^ Copyright 2018, Elsevier. “Pen‐writing”: Reproduced with permission.^[^ ^117^ ^]^ Copyright 2018, Royal Society of Chemistry. “Physical vapor deposition”: Reproduced with permission.^[^ ^48^ ^]^ Copyright 2015, Wiley‐VCH. “Surface mounting”: Reproduced with permission.^[^ ^118^ ^]^ Copyright 2014, Wiley‐VCH. “Mixing”: Reproduced with permission.^[^ ^15^ ^]^ Copyright 2018, Wiley‐VCH.

When applying the physical methods to construct electronics on flexible porous substrates, functional materials are integrated physically without generating new substances. As shown in **Figure** **10**, common physical methods include printing, coating, pen‐writing, physical vapor deposition (PVD), surface mounting, and mixing. To select the appropriate functionalization method, many factors should be considered, including substrate characteristics, fabrication resolution, process speed, thickness control, and cost.^[^ ^7^ ^]^ In this section, we briefly introduce the basic working mechanism, key process parameters, and manufacturing process of various physical methods. Their advantages/disadvantages and typical conductive materials used are summarized in **Table** **2**.

**Table 2:** Summary of advantages/disadvantages of physical methods and typical conductive materials used

Physical method		Typical conductive materials	Advantages	Disadvantages	Substrates
Printing	Inkjet printing	Ag nanoparticles,^[^ ^119^ ^]^ CuO,^[^ ^120^ ^]^ PEDOT:PSS,^[^ ^121^ ^]^ graphene, CB^[^ ^122^ ^]^	High‐resolution complex patterns, cost‐effectiveness, high adaptivity with different inks, low material consumption	Limited printing speed, nozzle clogging, droplet uneven drying	Almost no limitations
	Screen printing	Ag NWs,^[^ ^123^ ^]^ Ag nanoflakes,^[^ ^70^ ^]^ graphene, CB, GO^[^ ^115^ ^]^	Compatible with a wide range of ink formulations, low resistance thick layers, industrial scalable	Time‐consuming and expensive screen plate preparation	Flat porous substrates
Coating	Casting	CB,^[^ ^124^ ^]^ SnS_2_/RGO^[^ ^125^ ^]^	Low cost and simplicity	Inaccurate control of coverage area and film thickness	Almost no limitations
	Dip‐coating	PANI,^[^ ^42^ ^]^ Ag NWs,^[^ ^110^ ^]^ RGO, CNTs,^[^ ^126^ ^]^ carbon nanofibers^[^ ^127^ ^]^	Simple, low‐cost, uniform thin layer with a large coverage area	Elaborate procedures and environmentally unfriendly chemicals, a lot of material waste	
	Spray coating	MWCNT,^[^ ^116^ ^]^ Ag NWs^[^ ^128^ ^]^	Cheap, simple, high material usage	Poor repeatability with multiple spraying cycles	
Pen‐writing	–	Graphite,^[^ ^129^ ^]^ Ag,^[^ ^130^ ^]^ RGO,^[^ ^131^ ^]^ Ti_3_C_2_^[^ ^132^ ^]^	Facile and low‐cost “on‐demand” production, compatible with curved substrates	Tedious optimization of writing and ink parameters	Paper, fabric, electrospun nanofibers
PVD	Sputtering	Ag, ^[^ ^133^ ^]^ Au, Cr, ^[^ ^134^ ^]^ Bi_2_Te_3_, ^[^ ^68^ ^]^ Tin‐doped indium oxide^[^ ^135^ ^]^	Mass production, good film adhesion, composite films	Contamination caused by uncontrolled atoms diffusion	Almost no limitations
	Thermal evaporation	Au,^[^ ^45^ ^]^ Al^[^ ^136^ ^]^	Simple and cheap devices	Not suitable for high melting point materials	
	Electron beam evaporation	Au, Ti,^[^ ^50^ ^]^ Cr^[^ ^48^ ^]^	High deposition rate and material usage, good film adhesion	Complicated and expensive devices, possible substrate damage by the generated X‐rays	
Surface mounting	–	–	Compatible with different types of off‐the‐shelf electronics	Intricate manual assembly, partial loss of substrate flexibility due to rigid components	Almost no limitations
Mixing	–	MWCNTs,^[^ ^137^ ^]^ carbon fibers,^[^ ^138^ ^]^ PPy,^[^ ^139^ ^]^ PEDOT:PSS^[^ ^140^ ^]^	Fiber/molecular level integration, tightly bonding of conductive material	Complex manufacturing processes, the multifaceted impact of adding functional materials	Exclude natural porous substrates

#### 3.1.1 Printing

Printing technology has a long history. In the past, it described a method of transferring a layer of ink to a substrate through a stamp.^[^ ^141^ ^]^ Nowadays, the printing technique has mainly developed into two branches: digital printing and non‐digital printing. Digital printing (e.g., inkjet printing, aerosol jet printing, electrohydrodynamic printing) can directly convert digital‐based images into the motion of a motorized stage to print diverse patterns on the substrate. It follows the drop‐on‐demand fabrication style, with minimized material waste and high resolution,^[^ ^4b^ ^]^ while non‐digital printing (e.g., screen printing, stencil printing, microcontact printing) still uses the original template‐based working mechanism, creating patterns with predefined masks, stencils, screens, or soft stamps.^[^ ^142^ ^]^

Among different digital printing methods, inkjet printing exhibits great advantages in fabrication cost and scalability. Generally, the inkjet printer is made up of a print head with micrometer size nozzles and an ink reservoir. The ink droplets can be ejected out of the nozzle in continuous mode or drop‐on‐demand mode driven by a thermal excitation or piezoelectric effect. As a non‐contact printing technology, inkjet printing is suitable for a variety of porous substrates without being affected by the surface structure. Besides, it is compatible with many functional materials (e.g., silver nanoparticles,^[^ ^119^ ^]^ copper oxide,^[^ ^120^ ^]^ PEDOT:PSS,^[^ ^121^ ^]^ graphene, CB,^[^ ^122^ ^]^ and their composites^[^ ^143^ ^]^), and can fabricate various electronic components including conductive patterns,^[^ ^144^ ^]^ antenna,^[^ ^65^ ^]^ transistor,^[^ ^145^ ^]^ sensor,^[^ ^146^ ^]^ and SC.^[^ ^62^ ^]^ The printing quality such as resolution, ink penetration depth, and conductivity can be controlled by adjusting the ink parameters (e.g., formulation, surface energy, temperature, rheological properties), the nozzle diameter, and properties of the substrate.^[^ ^147^ ^]^

**Figure 11:**
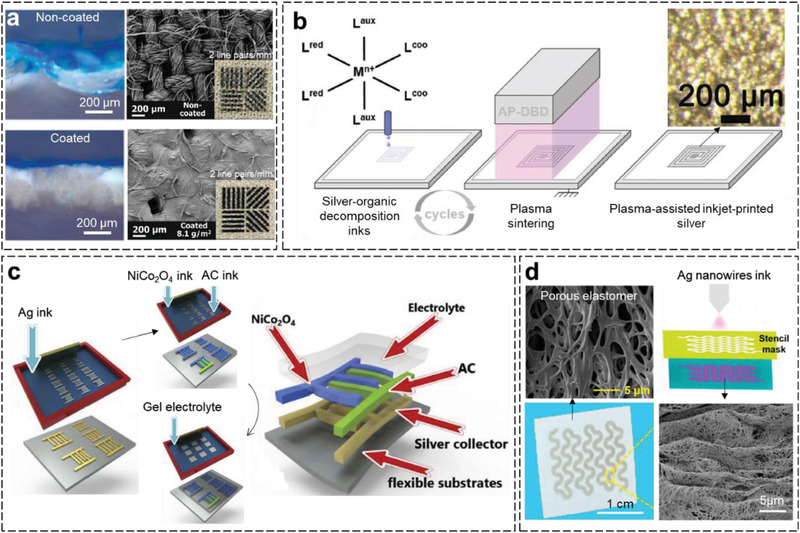
Examples of employing printing and coating techniques to functionalize flexible porous substrates. a) Improving the inkjet printing resolution on fabric by CNF coating. Reproduced with permission.^[^ ^144^ ^]^ Copyright 2017, American Chemical Society. b) Room‐temperature plasma‐assisted inkjet printing. Reproduced with permission.^[^ ^149^ ^]^ Copyright 2018, Wiley‐VCH. c) Systematical three‐step screen printing technique. Reproduced with permission.^[^ ^152^ ^]^ Copyright 2019, Elsevier. d) Using spray coating on multiscale porous elastomer to fabricate multifunctional on‐skin electronics. Reproduced with permission.^[^ ^154^ ^]^ Copyright 2020, National Academy of Sciences.

Uncontrolled ink diffusion and penetration are common problems in inkjet printing on porous substrates, which greatly limits print quality. These challenges can be addressed by optimizing the surface properties of the porous substrate. For example, CNFs have been used to optimize the porous surfaces of A4 paper,^[^ ^62^ ^]^ cardboard,^[^ ^148^ ^]^ and woven cotton fabrics.^[^ ^144^ ^]^ As displayed in **Figure** **11**a, the printing resolution is significantly improved after the surface modification because the CNF coating effectively limits the diffusion and penetration of the ink on the porous substrate.^[^ ^144^ ^]^ In addition, the limited conductivity of printed circuits on porous substrates is another problem. Apart from changing the composition and concentration of the conductive ink, modified inkjet printing technology has been invented to solve this problem. Figure 11b presents a room‐temperature plasma‐assisted inkjet printing technique reported by Knapp et al.^[^ ^149^ ^]^ for the deposition of highly conductive silver features on paper. This atmospheric plasma sintering method ensures the immediate conversion of silver‐organic decomposition inks after printing, avoiding ink wetting/penetration and thermal treatment. Therefore, it is particularly suitable for the coating of porous and temperature‐sensitive substrates such as paper.

Non‐digital printing techniques such as screen printing, gravure printing, and flexography printing are industrial scalable. Among them, screen printing using low‐cost masks made of fabric or stainless steel is the cheapest one.^[^ ^147^ ^]^ During the printing process, the ink is pushed through the woven mesh by a blade or squeegee. Subsequently, the ink is extruded from the open mesh and is impermeable in the blocking stencil areas, thus forming ink patterns on the substrate.^[^ ^129^ ^]^ Generally, inks with high viscosity and shear thinning characteristics are utilized because low‐viscosity inks can easily pass through the mesh and mess up the pattern. The printing resolution and thickness are mainly determined by the ink properties, mesh density, and surface properties of the substrate. Because of its simplicity, high speed, and wide applicability, screen printing has been applied on diverse porous substrates (e.g., paper, fabric, and electrospun nanofibers) to fabricate low‐cost SC, RFID tags,^[^ ^150^ ^]^ and sensors.^[^ ^151^ ^]^ Electronic components with different ink compositions can be printed layer by layer through appropriate structural design. For instance, Liu and co‐authors^[^ ^152^ ^]^ built in‐plane solid‐state SCs on paper and textile by a systematical three‐step screen printing technique as shown in Figure 11c. Silver collectors with different structural designs were printed on the porous substrate first. Then, NiCo_2_O_4_ nanowires and active carbon (AC) were printed as positive and negative electrodes. Finally, the gel electrolyte (PVA‐KOH) was printed on the electrodes to fabricate asymmetric SCs.

#### 3.1.2 Coating

“Coating” is the process of transferring the ink to the substrate using actions such as pouring, painting, spraying, casting, and smearing. Unlike printing methods that produce complex patterns, coating techniques are often used to create a uniform film on the entire substrate. Casting, dip coating, and spray coating are three typical coating techniques to functionalize porous substrates. Casting is the simplest film‐forming technology. By casting the functional material suspension, the substrate can be functionalized after solvent drying without any additional equipment. For example, active inorganic materials (e.g., CB,^[^ ^124^ ^]^ SnS_2_/RGO heterojunction)^[^ ^125^ ^]^ were drop‐casted on paper and porous liquid crystal polymer (LCP) substrate to construct electrodes for low‐cost sensors.

Dip coating and vacuum filtration are very effective methods for infiltrating conductive materials into porous substrates. In the manufacturing process, the porous substrate is first immersed in a solution containing functional materials, and then the substrate is removed, or the solution is drained at a controlled rate. The coating thickness is mainly controlled by the immersion time, the withdrawal speed, the number of coating cycles, properties of solution (e.g., viscosity, concentration), etc.^[^ ^25^, ^147^ ^]^ A variety of porous substrates such as textile, leather, PDMS sponge, and PU sponge have been dip‐coated with different functional materials (e.g., PANI, Ag NWs, RGO, CNTs, carbon nanofibers) to produce different sensors.

Spraying coating uses an atomizer to force the low‐viscosity solution through the nozzle to form fine droplets, which are then accelerated toward the substrate by an inert carrier gas. This method is very suitable for making large‐area conductive films on non‐planar porous substrates. The thickness and uniformity of the coating can be controlled by the spray speed, the number of sprayed layers, the distance between substrate and spray nozzle, and the droplet size.^[^ ^25a^ ^]^ Numerous porous substrates including paper,^[^ ^38^ ^]^ cotton fabric,^[^ ^116^ ^]^ electrospinning PU textile,^[^ ^128^ ^]^ and porous PDMS^[^ ^153^ ^]^ have been functionalized by spray coating to fabricate electrodes for TENG and various sensors. As shown in Figure 11d, Ag NWs were spray‐coated on a porous elastomer to construct multifunctional on‐skin electronics for sensing electrophysiological signals, temperature, hydration, and pressure.^[^ ^154^ ^]^ The multi‐scale porous elastomer can passively cool human bodies without consuming any additional energy.

#### 3.1.3 Pen‐Writing

The pen‐writing method can transfer and pattern conductive materials on the substrate via simple writing actions, which is especially suitable for the “on‐demand” production of paper electronics. Only using ordinary office supplies, various electronic components can be designed and produced within a few minutes. Based on different drawing materials, the pen‐writing method can be divided into “pencil drawing” and “do‐it‐yourself (DIY) pen writing”.

Commercial pencil fillers are made by mixing graphite and clay in a certain proportion. By drawing on paper with a pencil, graphite circuits and electrodes can be easily made in a fast and solvent‐free manner. These graphitic tracks are mechanically robust, lightweight, environmentally friendly, and resistant to chemical corrosion, heat, and radiation.^[^ ^129^ ^]^ In recent years, different types of electronics such as humidity sensors,^[^ ^66^ ^]^ pressure sensors,^[^ ^117^ ^]^ and TENGs^[^ ^155^ ^]^ have been generated by pencil drawing on paper substrates. The resulting porous graphite‐paper fiber hybrid structures can work as passive conductive electrodes and active sensing materials. Their conductivity mainly depends on the graphite content, paper roughness, and drawing parameters.

**Figure 12:**
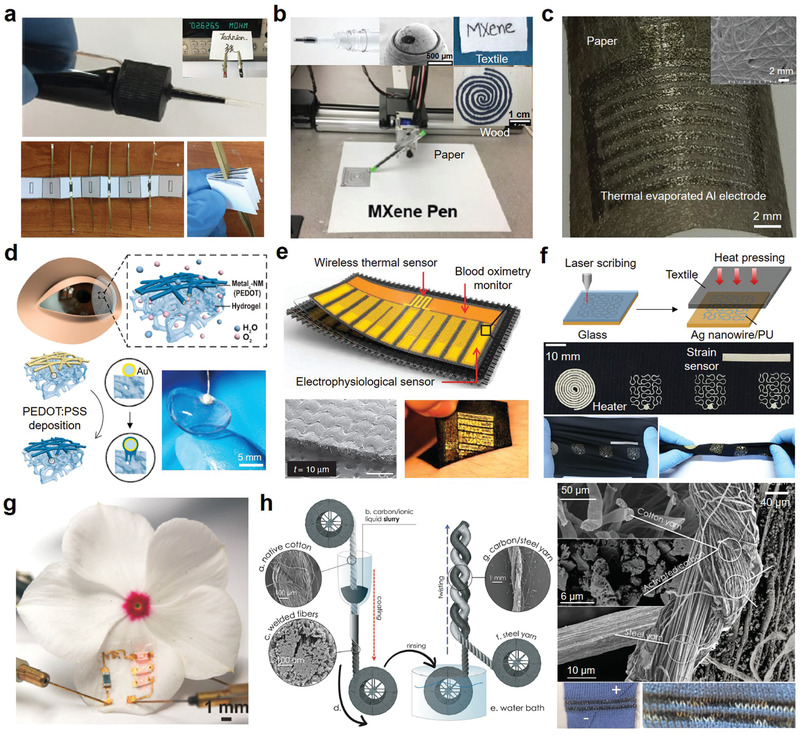
Examples of using pen‐writing, PVD, surface mounting, and mixing techniques to functionalize flexible porous substrates. a) Origami hierarchical electronics fabricated by a vial‐based DIY pen. Reproduced with permission.^[^ ^131^ ^]^ Copyright 2019, Springer Nature. b) A rollerball pen containing Ti_3_C_2_ ink installed on the AxiDraw setup to realize computer‐controlled automatic drawing. Reproduced with permission.^[^ ^132^ ^]^ Copyright 2018, Wiley‐VCH. c) The interdigitated Al electrode manufactured by thermal evaporation on the GO‐coated paper. Reproduced with permission.^[^ ^136^ ^]^ Copyright 2019, Elsevier. d) An electroretinogram sensor manufactured by exploiting PEDOT:PSS to bond Au‐coated nanofiber mesh to the porous hydrogel contact lens. Reproduced with permission.^[^ ^160^ ^]^ Copyright 2019, American Chemical Society. e) A fabric‐based multifunctional on‐skin sensor developed by transfer printing. Reproduced with permission.^[^ ^39^ ^]^ Copyright 2014, Springer Nature. f) Multifunctional e‐textiles produced by heat pressing. Reproduced with permission.^[^ ^162^ ^]^ Copyright 2019, American Chemical Society. g) CNF‐based conformal electronic decal transferred on a flower petal. Reproduced with permission.^[^ ^163^ ^]^ Copyright 2014, Wiley‐VCH. h) Textile SCs are achieved by knitting conductive composite yarns into stretchable fabrics. Reproduced with permission.^[^ ^79^ ^]^ Copyright 2014, Wiley‐VCH.

The DIY pen writing modifies commercially available rollerball pens, fountain pens, brush pens, etc., and employs customized inks on the porous substrates. The quality of writing is governed by the characteristics of the substrate (e.g., surface roughness, textures, bending stiffness), the properties of the ink (e.g., compositions, viscosity, rheological properties), and the drawing process (e.g., drawing directions and cycle numbers). Various functional devices can be developed on paper and fabrics by DIY pen writing. For example, **Figure** **12**a shows a vial‐based DIY pen containing RGO/polydopamine ink. Using this pen, Zhang et al.^[^ ^131^ ^]^ designed a time‐space‐resolved origami hierarchical sensor array, which can sense and distinguish temperature, humidity, light, and volatile organic compounds simultaneously. Handwriting is suitable for creating simple patterns, but it cannot create complex patterns quickly and efficiently. To solve this problem, Gogotsi's group^[^ ^132^ ^]^ installed a rollerball pen containing Ti_3_C_2_ ink on the AxiDraw setup to realize computer‐controlled automatic drawing as shown in Figure 12b. SCs and conductive traces with complex predesigned patterns have been successfully written on paper, textile, and wood.

#### 3.1.4 Physical Vapor Deposition

PVD refers to various vacuum deposition methods in which the target materials first transform from the condensed phase to the vapor phase, and then travel to the substrate and condense to form a solid thin film. They usually require expensive equipment but are still indispensable approaches for manufacturing high‐performance electronics on porous substrates. Next, we will discuss the two representative PVD methods, sputtering and evaporation deposition.

During sputtering, the “target” material is bombarded by a glow plasma discharge, which is generally localized around the “target” by a magnet. Atoms or molecules near the “target” surface will escape from the solid surface after obtaining sufficient energy and sputter onto the substrate in the form of vapor to form a thin film. The thickness and quality of the film are affected by sputtering process parameters (e.g., pressure, power, time) and substrate temperature. Almost all metals, alloys, and ceramic materials can be prepared as targets, and multi‐target co‐sputtering can deposit alloys in precise and constant proportions under appropriate conditions. For example, metals (i.e., Ag, Au, and Cr) have been sputtered on PU sponge, electrospun PVA nanofilm, and porous LCP substrate to produce conductors^[^ ^156^ ^]^ and sensors.^[^ ^133^, ^134^ ^]^ In addition, alloys (i.e., Bi_2_Te_3_)^[^ ^68^ ^]^ and metallic oxide (i.e., Tin‐doped indium oxide)^[^ ^135^ ^]^ have been sputtered onto paper to make high‐performance thermoelectric materials and solar cell electrodes, respectively.

In evaporation deposition, the source material is heated and evaporated in a vacuum. Subsequently, the vapor particles diffuse to the substrate and condense into a solid thin film. Compared with sputtering, evaporation has a higher deposition rate and less impact on the substrate, but sputtering generally exhibits better step coverage and accurate control of film thickness and uniformity. The evaporation method has been classified into various categories based on the heating sources. Thermal evaporation and electron beam evaporation are two typical evaporation methods. The former uses electrical resistance to heat the source material to high vapor pressure, while the latter uses electron bombardment to heat the source material. The thickness and uniformity of the deposited film are mainly controlled by the vacuum, evaporation time, substrate roughness, and the distance between source material and substrate. They are good at building metal electrodes (e.g., Au, Ti, Cr, Al) on porous substrates for diverse sensors.^[^ ^45^, ^48^, ^50^, ^136^ ^]^ Figure 12c shows the interdigitated Al electrode manufactured by thermal evaporation on the GO‐coated paper, which has a good response toward humidity with low hysteresis (the maximum hysteresis of 2.85% at 60% RH remained the same for all humidity exposure cycles).^[^ ^136^ ^]^

#### 3.1.5 Surface Mounting

Surface mounting refers to the integration of commercial integrated circuits/electrodes, off‐the‐shelf miniaturized electronics, or film‐based electronic components on porous substrates. Generally, the electronic components and the substrate are bonded by a conductive adhesive layer (e.g., tapes, glues, and other adhesives). Hence, the fabrication quality is related to the device assembly process and the bonding strength between different layers. Surface mounting is particularly suitable for assembling batteries^[^ ^157^ ^]^ and TENGs^[^ ^75^, ^158^ ^]^ with laminated structures. For example, to produce paper‐based flexible batteries, Wang et al.^[^ ^157^ ^]^ used a double‐conductive Cu tape to attach Al anode to the paper‐based gel electrolyte, in which the Cu tape also served as the anode current collector. By attaching Cu foils to paper and porous polytetrafluoroethylene (PTFE) film with conductive adhesives, Mao et al.^[^ ^158^ ^]^ assembled a paper‐based TENG. In addition to conductive adhesives, silver paste has been used to fix commercially available electrodes on a porous ionic membrane to produce flexible humidity sensors.^[^ ^159^ ^]^ As shown in Figure 12d, by bonding Au‐coated nanofiber mesh to the porous hydrogel contact lens with PEDOT:PSS, Duan's group^[^ ^160^ ^]^ produced an electroretinogram sensor for eye interfacing.

Transfer printing is another effective surface mounting technology, in which thin‐film‐based electronics are first released from an intermediate substrate coated with a sacrificial layer and then transferred to the target substrate. Silicon wafer is often used as the intermediated substrate to facilitate the production of thin‐film‐based electronics. After fabrication, the constructed devices are released by dissolving the sacrificial layer. For example, to create stretchable electronics, serpentine circuits were released from the silicon wafer by dissolving the poly(methyl methacrylate) layer with acetone and then transferred to various porous substrates including paper, leather, and fabric.^[^ ^161^ ^]^ Glass slides can also be used as the intermediated substrate and water‐soluble tapes are usually served as the sacrificial layer. Employing this approach, stretchable epidermal sensors have been achieved on textile,^[^ ^39^ ^]^ cellulose paper, and various sponges made of cellulose, PU, PVA, and silicone.^[^ ^118^ ^]^ Figure 12e illustrates the fabric‐based multifunctional on‐skin sensor developed by Rogers's group^[^ ^39^ ^]^ with transfer printing, which can measure hydration state, electrophysiological activity, pulse, and cerebral oximetry. Using heat pressing, Yao et al.^[^ ^162^ ^]^ successfully transferred multiple electronic components (i.e., electrodes, strain sensor, heater) from the glass substrate to the stretchable fabric (Figure 12f). With the help of water‐soluble sugar (polysaccharide), Daniele et al.^[^ ^163^ ^]^ built CNF‐based conformal electronic decals, which can be easily transferred to diverse biological substrates. Figure 12g demonstrates the LED circuit decal adhered to a flower petal by static forces.

#### 3.1.6 Mixing

The “mixing” method can functionalize the porous substrate at the fiber level or even the molecular level by adding conductive materials to its raw materials or precursor solutions during the substrate fabrication process. It is suitable for both fiber‐based and non‐fiber‐based porous substrates. For instance, conductive papers have been achieved by mixing conductive materials (e.g., MWCNTs,^[^ ^137^ ^]^ carbon fibers,^[^ ^138^ ^]^ PPy,^[^ ^139^ ^]^ PEDOT:PSS,^[^ ^140^ ^]^ poly (4‐styrenesulfonic acid))^[^ ^164^ ^]^ in the cellulose pulp suspensions. The resulting conductive papers can be applied to actuators, sensors, and SCs, serving as substrates, sensing materials, electrodes, and electrolytes. By assembling conductive fiber electrodes with different functions, Wang's group^[^ ^165^ ^]^ fabricated fiber‐shaped nanogenerators, solar cells, and SCs. By knitting these energy devices, self‐powered textiles have been achieved. Similarly, Jost et al.^[^ ^79^ ^]^ designed textile SCs by knitting conductive composite yarns into stretchable fabrics. As displayed in Figure 12h, the composite yarns were obtained by twisting carbon/ionic liquid‐coated cotton fibers and steel yarns. Besides, Cao et al.^[^ ^166^ ^]^ generated stretchable textiles by 3D printing with hybrid inks made of oxidized CNFs and Ti_3_C_2_ for strain sensing.

For non‐fiber‐based porous substrates such as PU and PDMS sponges, conductive nanomaterials (e.g., graphene,^[^ ^13^, ^167^ ^]^ CNTs,^[^ ^15^, ^51^, ^108^ ^]^ carbon nanofibers^[^ ^127^ ^]^) can be dispersed in their prepolymer solution and uniformly distributed in the substrate after curing. Because of their excellent flexibility and deformability, these obtained composite sponges are commonly used as strain or pressure sensors. In addition, nanomaterials coated with conductive materials can also be incorporated. For example, PPy‐coated cellulose nanocrystals and nanofibers have been added into PVA solution to produce a porous nanocomposite for self‐healable strain sensor.^[^ ^168^ ^]^

### 3.2 Chemical Methods

**Figure 13:**
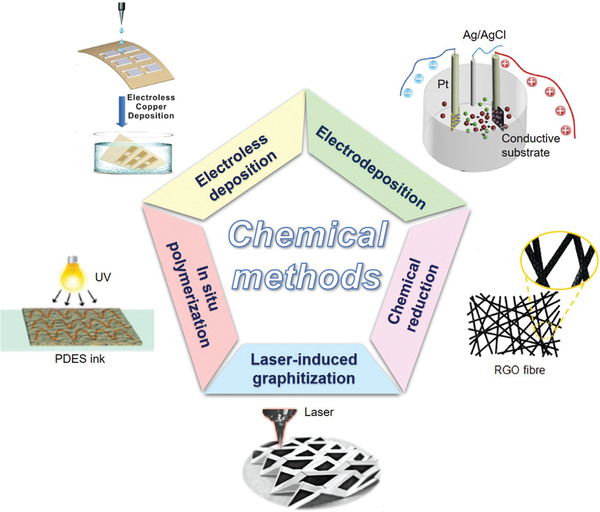
Chemical methods for the functionalization of flexible porous substrates. “Electroless deposition”: Reproduced with permission.^[^ ^35^ ^]^ Copyright 2018, Wiley‐VCH. “Electrodeposition”: Reproduced with permission.^[^ ^169^ ^]^ Copyright 2018, Elsevier. “Chemical reduction”: Reproduced with permission.^[^ ^170^ ^]^ Copyright 2013, Wiley‐VCH. “Laser‐induced graphitization”: Reproduced with permission.^[^ ^171^ ^]^ Copyright 2018, Wiley‐VCH. “In situ polymerization”: Reproduced with permission.^[^ ^172^ ^]^ Copyright 2018, Royal Society of Chemistry.

In chemical methods, chemical reactions such as redox reactions, catalytic reactions, and polymerizations are involved, and new substances are produced during the substrate functionalization. **Figure** **13** exhibits the chemical methods for the functionalization of flexible porous substrates. Employing these methods, various conductive materials, such as metals, carbon‐based materials, and conductive polymers, can be grown in situ on porous substrates. For example, copper, nickel, and gold can be deposited by ELD and electrodeposition. Graphene, CNTs, and their composites can be produced by chemical reduction and laser‐induced graphitization (LIG). PANI and PPy can be prepared by in situ polymerization and electrodeposition. Chemical methods are usually combined with physical methods to improve the mechanical properties and conductivity of the substrate. To obtain the expected active substances with controlled structures, it is essential to understand the reaction processes and basic characteristics of each chemical method. In this part, the reaction mechanism, dominant reaction parameters, and fabrication processes of each chemical method are briefly described. Their advantages/disadvantages and typical conductive materials used are enumerated in **Table** **3**.

**Table 3:** Summary of advantages/disadvantages of chemical methods and typical conductive materials used

Chemical method	Typical conductive materials	Advantages	Disadvantages	Substrates
ELD	Ni,^[^ ^83^ ^]^ Cu,^[^ ^173^ ^]^ Au, Ag, Pt^[^ ^174^ ^]^	Low‐cost and environmentally friendly, uniform and compact coatings with good corrosion resistance and strong adhesion, no DC power required	Complex and unstable ELD solution, continuous component analysis and supplement, slower deposition rate	Almost no limitations
Electrodeposition	Au,^[^ ^175^ ^]^ Co(OH)_2_,^[^ ^176^ ^]^ MnO_2_,^[^ ^177^ ^]^ Mn_3_O_4_,^[^ ^178^ ^]^ PPy,^[^ ^179^ ^]^ PEDOT:PSS^[^ ^160^ ^]^	Wide range of applicable materials (metals, alloys, metal oxides, polymers, composites), fast deposition rate	Only applicable for conductive surfaces	Conductive porous substrates
Chemical reduction	RGO^[^ ^102^ ^]^	Convenient, low‐cost, and commercially scalable, little impact on the original 3D structure	Defects in RGO and lower conductivity than pristine graphene	Almost no limitations
LIG	Molybdenum carbide‐graphene,^[^ ^171^ ^]^ laser‐induced graphene^[^ ^180^ ^]^	Very promising for commercial applications, no mask required	Defects and impurities of laser‐induced graphene	Biodegradable porous substrates
In situ polymerization	PPy,^[^ ^181^ ^]^ PANI,^[^ ^182^ ^]^ PEDOT^[^ ^183^ ^]^	Increasing the conductivity and mechanical strength of porous substrates, tight binding of conductive polymer	Flammable and explosive chemicals (monomers, solvents, initiators, catalysts), unstable structure of the polymer	Almost no limitations

#### 3.2.1 Electroless Deposition

**Figure 14:**
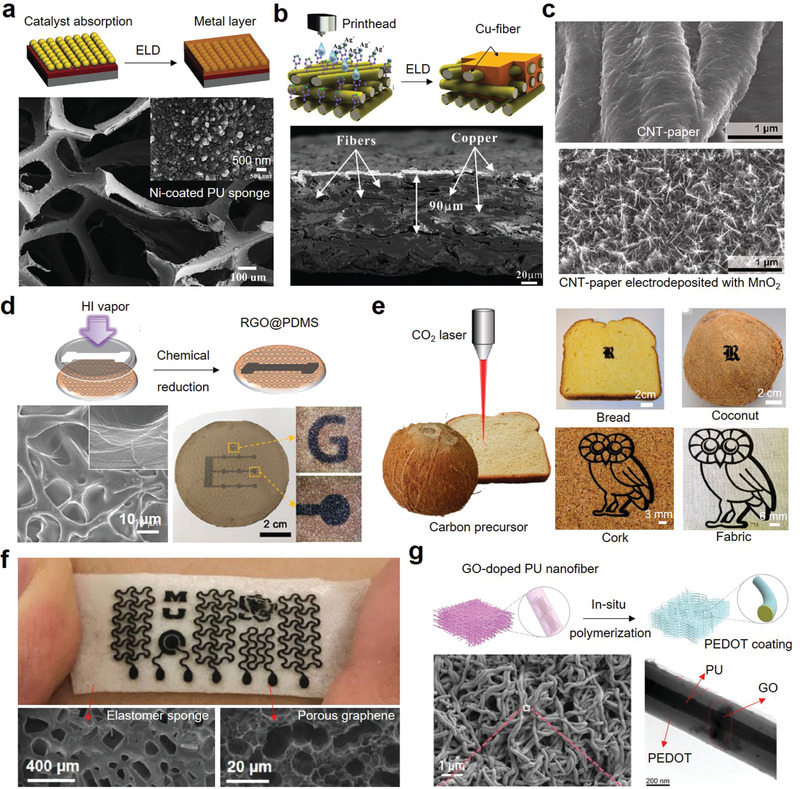
Examples of employing chemical methods to functionalize flexible porous substrates. a) Highly flexible metalized PU sponge prepared by Ni ELD. Reproduced with permission.^[^ ^83^ ^]^ Copyright 2019, Wiley‐VCH. b) Hybrid copper‐fiber conductive structures fabricated by inkjet printing and Cu ELD. Reproduced with permission.^[^ ^173^ ^]^ Copyright 2017, Wiley‐VCH. c) The surface morphology of CNT‐coated paper before and after MnO_2_ electrodeposition. Reproduced with permission.^[^ ^177^ ^]^ Copyright 2013, American Chemical Society. d) Highly elastic porous RGO‐PDMS films produced by the low‐temperature HI vapor reduction. Reproduced with permission.^[^ ^19^ ^]^ Copyright 2017, Wiley‐VCH. e) The graphene patterns on bread, coconut shells, cork, and fabric implemented by LIG. Reproduced with permission.^[^ ^180^ ^]^ Copyright 2018, American Chemical Society. f) Multifunctional on‐skin electronics fabricated on silicone elastomer sponges by LIG and transfer printing. Reproduced with permission.^[^ ^190^ ^]^ Copyright 2018, Wiley‐VCH. g) The elastic GO‐doped PU nanofiber membrane functionalized by in situ PEDOT polymerization. Reproduced with permission.^[^ ^191^ ^]^ Copyright 2017, American Chemical Society.

Based on controllable redox reaction and metal catalysis, ELD uses strong reducing agents to reduce metal ions into metal nanoparticles, forming a dense coating on the catalyst‐loaded area of the substrate without the need for an external power supply. Before ELD, the porous substrate is first activated by adsorbing a layer of catalyst solution containing noble metal compounds (e.g., AgNO_3_,^[^ ^173^ ^]^ PdCl_2_,^[^ ^176^ ^]^ (NH_4_)_2_PdCl_4_,^[^ ^83^ ^]^ H_2_PtCl_6_).^[^ ^58^ ^]^ The composition of the ELD solution includes metal salts, reducing agents, complexing agents, and buffering agents. If the porous substrate is fully immersed in the catalyst solution and activated, the entire substrate will be metalized by ELD. The metalized paper/fabrics can function as electrodes for SCs,^[^ ^184^ ^]^ lithium‐ion batteries,^[^ ^185^ ^]^ and TENGs.^[^ ^186^ ^]^ Because of the strong bond between the metal particles and porous substrates, these electrodes exhibit excellent electrochemical stability and conductivity. Apart from paper and fabrics, other porous substrates can also be metalized by ELD. **Figure** **14**a demonstrates a highly flexible metalized PU sponge prepared by Ni ELD for human touch and arm motion monitoring.^[^ ^83^ ^]^

Combining ELD with printing technology (e.g., screen printing and inkjet printing) can implement selective metallization of the substrate. Catalyst ink can be printed on the predefined areas on the substrate; then the ink patterned area will be metalized by ELD. Combining inkjet printing and Cu ELD, Yang's group^[^ ^173^ ^]^ created highly conductive hybrid copper–fiber structures as displayed in Figure 14b for the fabrication of low‐cost high‐performance paper electronics. This strategy is also applicable to fabric substrates. Employing wax printing and ELD, Grell et al.^[^ ^174^ ^]^ developed a binder‐free method for autocatalytic metallization of fabrics, which is applicable to multiple metals. The hydrophobic wax barriers were first produced by inkjet printing, and then the catalytical Si ink was cast in the predefined area. After immersing the activated fabrics in the corresponding ELD solutions, diverse functional metal patterns (i.e., Au, Ag, and Pt) can be obtained on the fabric.

#### 3.2.2 Electrodeposition

Electrodeposition uses an external electric field to drive the migration of positive and negative ions in the electrolyte and the redox reaction on the electrode to form a conductive coating. To better control the deposition process, electrodeposition is usually carried out under galvanostatic conditions in a three‐electrode cell connected to an electrochemical workstation. The three‐electrode system consists of a working electrode (i.e., part to be plated), a reference electrode (i.e., Ag/AgCl), and a counter electrode (i.e., platinum). The electrodeposition solution is utilized as the electrolyte. Because electrodeposition is only applicable for conductive substrates, it is generally combined with other approaches such as dip‐coating, mixing, ELD, or carbonization to further improve the conductivity and capacitance of paper/fabric electrodes for flexible SCs. A wide variety of conductive materials including metals (e.g., gold),^[^ ^175^ ^]^ metal hydroxides or oxides (e.g., Co(OH)_2_,^[^ ^176^ ^]^ MnO_2_,^[^ ^177^ ^]^ Mn_3_O_4_),^[^ ^178^ ^]^ and conductive polymers (e.g., PPy,^[^ ^179^ ^]^ PEDOT:PSS)^[^ ^160^ ^]^ have been electrodeposited on conductive paper/fabrics. For example, a cellulose paper was first dip‐coated with CNTs and then electrodeposited with MnO_2_ to manufacture hybrid SC electrodes.^[^ ^177^ ^]^ Figure 14c shows the surface morphology of CNT‐coated paper before and after MnO_2_ electrodeposition. The cellulose paper substrate offers a large surface area and can act as an interior electrolyte reservoir. The water swelling effect of cellulose fibers promotes the absorption of electrolytes, and the mesoporous internal structure of the fibers provides ion diffusion channels for electrochemical energy storage materials.

#### 3.2.3 Chemical Reduction

Chemical reduction uses chemical reagents to increase electrons or decrease the oxidation state of atoms, ions, or certain atoms in molecules. One typical example of using chemical reduction to functionalize porous substrates is the dip‐and‐reduction process, which converts GO coated on the substrate into RGO. The insulating GO is used because of its superior dispersibility in water. Adopting this strategy, diverse porous substrates including porous PDMS,^[^ ^102^ ^]^ PU sponge,^[^ ^109^ ^]^ 3D printed porous PVA,^[^ ^187^ ^]^ and fabrics^[^ ^170^ ^]^ have been functionalized to construct stretchable conductors, strain sensors, pressure sensors, and conductive wires. GO can be reduced into RGO by solution‐phase reduction methods using different reducing agents such as hydroiodic (HI) acid, hydrazine hydrate, l‐ascorbic acid, and NaBH_4_. Vapor phase reduction is another effective method to transfer GO into RGO. Utilizing the low‐temperature HI vapor reduction, Yun et al.^[^ ^19^ ^]^ fabricated highly elastic porous RGO‐PDMS films for epidermal strain sensors. As exhibited in Figure 14d, by exposing the porous PDMS to HI vapor through a metal mask, the GO in the exposed area can be selectively reduced to form RGO patterns. In addition, thermal reduction has been exploited to reduce GO coated on polyester fabrics to fabricate piezoresistive pressure sensors.^[^ ^188^ ^]^

#### 3.2.4 Laser‐Induced Graphitization

LIG refers to the carbonization process in which carbon precursors (e.g., polyimide, lignocellulosic materials) are directly converted to graphene by laser irradiation with minimal ablation under an ambient atmosphere.^[^ ^189^ ^]^ The LIG quality is mainly determined by the laser type, laser irradiation process, and the composition of the substrate. The CO_2_ laser with a wavelength of 10.6 µm is a commonly used laser source for LIG due to its high power and conversion efficiency.^[^ ^189^ ^]^ Other types of lasers such as UV femtosecond laser pulses are helpful to achieve a lower graphitization temperature because of their high repetition rate and high photon energy that could localize the heat into a small area by efficient light absorption.^[^ ^99^ ^]^ In terms of the composition of the substrate, many fiber‐based porous substrates including woods, leaves, papers, and fabrics contain lignin. The higher the lignin content, the higher the photoconversion rate, but for heat‐sensitive materials with high cellulose contents, such as papers and fabrics, fire retardants need to be added before the LIG process to inhibit the ablation in ambient air.

In summary, LIG provides an effective and fast approach to convert biodegradable porous substrates into green graphene electronic devices. For example, through direct patterning of CO_2_ laser, Zang et al.^[^ ^171^ ^]^ quickly generated conductive molybdenum carbide‐graphene composites rapidly on paper to manufacture gas sensors, energy harvesters, and SCs. Figure 14e demonstrates the graphene patterns implemented by LIG on bread, coconut shells, cork, and fabric for biodegradable and edible electronics.^[^ ^180^ ^]^ Moreover, combining LIG with transfer printing, Yan's group^[^ ^190^ ^]^ developed multifunctional on‐skin electronics on silicone elastomer sponges. The obtained devices including electrophysiological sensors, hydration sensors, temperature sensors, and joule‐heating elements are illustrated in Figure 14f.

#### 3.2.5 In Situ Polymerization

Polymerization is a chemical process to produce polymers, in which monomer molecules react with the addition of an initiator or under external heat or light stimulation to form polymer chains or 3D networks. In situ solution phase polymerization can effectively functionalize porous substrates because the monomer solution can penetrate the porous structure and uniformly modify the surface with conjugated polymer by forming hydrogen bonds with the substrate. The coated monomers such as pyrrole, aniline, and EDOT can be converted into conductive PPy, PANI, and PEDOT by oxidative polymerization using different oxidants. For example, FeCl_3_ has been used to initiate the polymerization of PPy and PEDOT on cellulose papers to fabricate electrodes for SCs^[^ ^181^ ^]^ and batteries.^[^ ^183^ ^]^ Figure 14g shows the GO‐doped elastic PU nanofiber membrane functionalized by in situ PEDOT polymerization using FeCl_3_ oxidant for pressure and strain sensing.^[^ ^191^ ^]^ After polymerization, the 3D porous network structure of the substrate is well preserved, and the nanofiber surface is conformally coated with PEDOT, thus providing a large deformation space and more contact sites. The sensor demonstrates high sensitivity (up to 20.6 kPa^−1^) and a broad sensing range (1 Pa to 20 kPa). Tsao et al.^[^ ^192^ ^]^ prepared a stretchable PPy film on porous PDMS by in situ polymerization with traditional oxidant ammonium persulfate (APS). Similarly, with the help of APS, PANI was polymerized on silver‐coated paper^[^ ^182^ ^]^ and porous chitosan film^[^ ^193^ ^]^ for SCs and bioelectronic patches.

## 4 Electronic Devices Based on Flexible Porous Substrates

Each type of flexible porous substrate has its unique properties, which helps to create electronic devices with different functions to meet various application requirements. For example, papers are suitable for making disposable low‐cost electronic devices.^[^ ^6^ ^]^ Fabrics and electrospun nanofibers are particularly useful for manufacturing wearable electronics.^[^ ^194^ ^]^ Natural materials are promising substrates for biocompatible electronics,^[^ ^28^, ^97^ ^]^ edible electronics,^[^ ^195^ ^]^ biodegradable electronics,^[^ ^196^ ^]^ and implantable electronics.^[^ ^197^ ^]^ Porous elastic polymers like PDMS and PU are often exploited to develop stretchable electronics^[^ ^198^ ^]^ and on‐skin electronics.^[^ ^199^ ^]^ According to different functions, electronic devices on porous substrates can be categorized into basic electronic components (i.e., circuits, electrodes, RFID tags), sensors, energy storage and conversion devices, and other applications (e.g., actuators, HMI, biomedical devices). Here, we mainly cite some representative examples to show the special functions brought by the unique properties of porous substrates to electronic devices.

### 4.1 Circuits, Electrodes, and Passive RFID Tags

**Figure 15:**
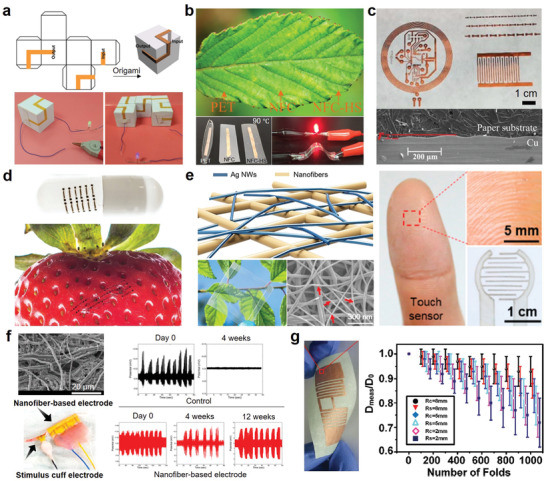
Circuits, electrodes, and passive RFID tags on flexible porous substrates with different functions for various application requirements. a) Origami 3D circuits. Reproduced with permission.^[^ ^210^ ^]^ Copyright 2018, Royal Society of Chemistry. b) Customized nanopaper made of thiol‐modified nanofibrillated cellulose with strong bonding with the Ag nanoparticle. Reproduced with permission.^[^ ^200^ ^]^ Copyright 2019, American Chemical Society. c) Highly conductive Cu‐paper circuits manufactured by a roll‐to‐roll method. Reproduced with permission.^[^ ^201^ ^]^ Copyright 2019, Institute of Physics Publishing Ltd. d) Edible tattoo paper‐based silver electrodes on pharmaceutical capsules and strawberries.^[^ ^205^ ^]^ Reproduced with permission. Copyright 2018, Wiley‐VCH. e) Invisible skin‐like electrodes on electrospun nanofibers. Reproduced with permission.^[^ ^206^ ^]^ Copyright 2018, American Chemical Society. f) Implantable electrospun polyimide nanofiber‐based electrode for neural signal recording. Reproduced with permission.^[^ ^207^ ^]^ Copyright 2017, American Chemical Society. g) Flexible RFID tag metal antenna prepared on a paper substrate and its normalized reading range (*D*_meas_/*D*_0_) versus bending times. Reproduced with permission.^[^ ^65^ ^]^ Copyright 2019, Wiley‐VCH.

Conductive circuits and electrodes are the basic components of various electronic devices. By printing conductive ink/paste, predesigned circuit patterns can be easily made on paper and fabrics. These printed circuits can be folded, bent, and stretched, exhibiting excellent flexibility. Taking the advantage of the good foldability of paper, Lu's group^[^ ^172^ ^]^ realized on‐demand input/output 3D circuits on a cellulose paper as displayed in **Figure** **15**a. The prepared 3D paper circuits show stable conductivity and robust mechanical properties and can be used in origami electronics. In addition to the mechanical strength, the biggest concern of printing circuits on porous substrates is the bonding strength of conductive materials and the substrate. To solve this problem, Yu et al.^[^ ^200^ ^]^ prepared a customized nanopaper made of thiol‐modified nanofibrillated cellulose (NFC‐HS) to improve the bond strength with the Ag nanoparticle ink. Due to the strong bond between the substrate and the ink, even after extensive peeling and bending, the printed circuit can maintain a stable conductivity of 2 × 10^−4^ Ω cm. As shown in Figure 15b, it shows high thermal dimensional stability and has transparency comparable to polyethylene terephthalate. Directly integrating metals on porous substrates is a more efficient way to create highly conductive circuits. Using a high‐speed roll‐to‐roll method, Li et al.^[^ ^201^ ^]^ directly laminated Cu and Al conductors on the paper substrate. Figure 15c demonstrates the obtained Cu‐paper circuits with low contact resistance (maximum 440 mΩ) and good mechanical reliability, which can withstand 9000 bending cycles without obvious changes in conductivity. Similarly, highly conductive fabric‐based circuits (6 × 10^6^ S m^−1^) have been achieved by printing gallium‐based semiliquid metals on cotton fabrics through roller printing technology.^[^ ^202^ ^]^

Electrodes based on flexible porous substrates with large specific surface areas, excellent deformability, and sufficient material exchange channels have received intense attention in the fabrication of high‐performance sensors and energy storage devices. Combining with various conductive materials, different functional electrodes have been made on papers and fabrics for wearable electronics such as SCs,^[^ ^203^ ^]^ strain sensors, TENGs,^[^ ^186^ ^]^ keyboards,^[^ ^204^ ^]^ and electrocardiography (ECG) sensors.^[^ ^121^ ^]^ In addition to wearability, other properties of porous substrates such as edibility, permeability, and biocompatibility open up a wide range of applications. For example, Bonacchini et al.^[^ ^205^ ^]^ developed organic edible electronics based on a versatile tattoo paper made of ethylcellulose. As shown in Figure 15d, the tattoo paper‐based silver electrodes have been successfully transferred to pharmaceutical capsules and strawberries. To produce invisible electronics, Fan and co‐authors^[^ ^206^ ^]^ produced skin‐like electrodes as illustrated in Figure 15e by embedding Ag NWs in the electrospun polyamide nanofibers. The electrode exhibits high optical transmittance (84.9% at 550 nm), good breathability (at a pressure drop of 300 Pa, air permeability of 6 cm^3^ s^−1^ cm^−2^ is achieved), and conformal bonding with skin. Because the substrate bears most of the load stress and maintains the connection between the Ag NWs, it shows low sheet resistance even after 3000 bending cycles. As demonstrated in Figure 15f, Kwon's group^[^ ^207^ ^]^ built an implantable electrospun polyimide nanofiber‐based electrode by inkjet printing for neural signal recording. After implantation, the electrode can record neural signals for a long period without nerve tissue shrinkage because the substrate allows the exchange of nutrients and oxygen to support cell viability.

RFID tags can communicate with the reader to identify the target in a non‐contact way. When the antenna of the passive RFID tag enters the electric field from the reader, it receives the radio frequency signal sent by the reader. Then an induced current is formed and powers the digital transponder circuit to send out the product information stored in the chip. RFID tags mainly work at 13.56 MHz, and the antenna is normally in the shape of a coil, similar in size to a credit card. To manufacture low‐cost flexible RFID tags, dipole antennas are usually printed on papers^[^ ^150^ ^]^ and fabrics^[^ ^208^ ^]^ using conductive ink made of silver or carbon‐based materials.^[^ ^209^ ^]^ The tight bond between the conductive inks and the porous substrates allows the antenna to maintain high mechanical flexibility and functionality after many deformation cycles. Figure 15g shows a flexible RFID tag metal antenna prepared on a paper substrate by inkjet printing and Cu ELD. The resulting metal antenna shows good adhesive strength and low resistivity of 2.58 × 10^−8^ Ω·m and maintains a reliable reading range after over 1000 bending cycles.^[^ ^65^ ^]^

### 4.2 Sensors

Based on different detection targets, the sensors are classified into physical sensors and chemical sensors. The former can convert the changes of physical quantities (e.g., force, temperature) into readable electrical signals. The latter is sensitive to various chemical substances and can convert their concentration changes into measurable electrical signals. Compared with traditional flat substrates, porous substrates can store more sensing materials and provide a larger surface area, helping to produce a variety of high‐performance physical sensors,^[^ ^211^ ^]^ chemical sensors,^[^ ^212^ ^]^ and hybrid multifunctional sensors.^[^ ^131^, ^213^ ^]^

#### 4.2.1 Physical Sensors

**Figure 16:**
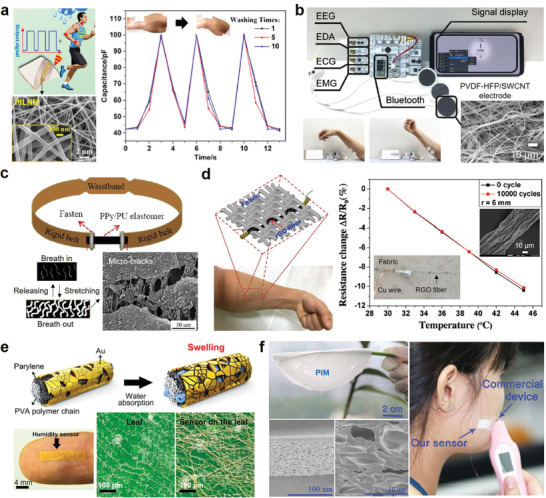
Physical sensors based on flexible porous substrates. a) Washable capacitive pressure‐sensing e‐textiles based on PILNM. Reproduced with permission.^[^ ^217^ ^]^ Copyright 2019, American Chemical Society. b) Blow‐spun nanofiber‐based multifunctional capacitive strain sensor. Reproduced with permission.^[^ ^219^ ^]^ Copyright 2019, Wiley‐VCH. c) Porous PU‐based highly stretchable strain sensor. Reproduced with permission.^[^ ^220^ ^]^ Copyright 2013, American Chemical Society. d) Freestanding RGO fiber‐based temperature sensor with a stable temperature response before and after 10 000 bending cycles. Reproduced with permission.^[^ ^221^ ^]^ Copyright 2018, Wiley‐VCH. e) Electrospun PVA nanofiber‐based breathable humidity sensor. Reproduced with permission.^[^ ^226^ ^]^ Copyright 2019, American Chemical Society. f) Highly selective humidity sensor based on a PIM made of PVA/KOH polymer gel electrolyte. Reproduced with permission.^[^ ^159^ ^]^ Copyright 2017, Wiley‐VCH.

Physical sensors based on flexible porous substrates are usually designed to detect pressure, strain, humidity, and temperature. Piezoresistive pressure sensors and can be easily manufactured by adding conductive materials to PDMS sponges, paper, and fabric. Generally, their resistance increases with the increase of applied pressure, and the sensitivity and detection limit of the sensors depend on the deformability of the substrate. Due to the excellent deformability of sponge structures, piezoresistive PDMS sponges functionalized by CNTs ^[^ ^214^ ^]^ and graphene^[^ ^167^ ^]^ are often used in HMIs,^[^ ^215^ ^]^ e‐skin, and human motion monitoring.^[^ ^216^ ^]^ Their sensitivity can be adjusted by changing the concentration of carbon materials and the porosity of PDMS. The capacitive sensor is another typical pressure sensor, which consists of a dielectric layer and parallel electrodes. The variation of its capacitance can reflect the magnitude of the external force. Porous electrospun nanofibers with high dielectric properties and robust mechanical performance are ideal capacitive‐based sensing materials. Using hydrophobic poly(ionic liquid) nanofibrous membrane (PILNM) as the sensing material, Wang et al.^[^ ^217^ ^]^ developed washable capacitive pressure‐sensing e‐textiles as shown in **Figure** **16**a. Owing to the porous nanofibrous structure and good dielectric performance of the PILNM, the prepared pressure sensor exhibits a low detection limit (20 Pa) and a fast response time (30 ms).

Human skin is highly stretchable with about 100% strain. Stretchable porous substrates such as papers,^[^ ^218^ ^]^ fabrics,^[^ ^219^ ^]^ and PU elastomer^[^ ^220^ ^]^ are very suitable for making on‐skin strain sensors for human joint and muscle motion monitoring. For example, by vertically stacking the dielectric blow‐spun poly(vinylidene fluoride‐*co*‐hexafluoropropylene) (PVDF‐HFP) nanofibers and single‐walled carbon nanotubes (SWCNTs) blended conductive fabric, Ho et al.^[^ ^219^ ^]^ designed multifunctional all‐fabric capacitive strain sensors. The stacked fabric sensor shows a high gauge factor of over 130 and excellent mechanical durability. As shown in Figure 16b, it can record ECG, electromyogram (EMG), electroencephalogram (EEG), and electrodermal activity (EDA). In addition, Wang's group produced a highly stretchable strain sensor for human breath detection by functionalizing the porous PU with in situ PPy polymerization. The resulting PU sensor shows excellent stretchability (420% elongation) and small resistance (8.364 Ω·cm).^[^ ^220^ ^]^ Figure 16c exhibits the strongly bonded PPy/PU interface, which forms netlike microcracks under stretching, and achieves reversible conductivity changes during stretching and release cycles.

Body temperature can reflect human health. Fabric‐based wearable temperature sensors can monitor body temperature in real‐time. Figure 16d shows a freestanding RGO fiber‐based temperature sensor created by Lee's group.^[^ ^221^ ^]^ It can be easily integrated into garments such as socks and undershirts to sense skin temperature. It showed a fast response (7 s) and recovery time (20 s) to temperature change in the range of 30–80 °C and maintains a stable temperature response after 10 000 bending cycles. The device's thermal index can be tuned by changing the RGO reduction time. Interestingly, using thermoelectric materials, body temperature can be utilized to power wearable sensors. Jung et al.^[^ ^222^ ^]^ developed wearable self‐powered temperature sensors based on highly stretchable thermoelectric fabrics. Because the thermoelectric ink can penetrate the fabric and fully cover the individual fibers, the resulting sensor exhibits excellent durability up to 800 cycles of 20% strain and linear temperature sensing response across a wide strain range.

The humidity sensor is essential for controlling life systems and monitoring industrial production processes. Due to the hygroscopicity, cellulose papers are favorable sensing materials for low‐cost humidity sensors.^[^ ^223^ ^]^ The paper resistance decreases drastically after adsorbing the water molecules. For instance, by assembling an A4 printing paper with conductive adhesive tapes, Duan et al.^[^ ^224^ ^]^ constructed a humidity sensor for the monitoring of respiratory rate, baby diaper wetting, and skin humidity. Owing to the moderate hydrophilicity of the paper and the rational sensor structure design, it shows a good linear humidity sensing response within the humidity range from 41.1% to 91.5% RH. Other porous substrates including fabrics,^[^ ^225^ ^]^ electrospun nanofibers,^[^ ^226^ ^]^ porous ionic membrane (PIM),^[^ ^159^ ^]^ and porous LCP^[^ ^227^ ^]^ have also been used to fabricate flexible humidity sensors for respiration and skin humidity monitoring. Humidity sensing elements can be readily produced by integrating carbon‐based materials (GO, RGO) or metals (Ni, Au, Ag, Pt) into these porous substrates. When exposed to increased RH, these sensing elements show positive or negative conductance changes based on different sensing mechanisms. For example, Jeong et al.^[^ ^226^ ^]^ fabricated a breathable humidity sensor by depositing parylene C and gold on the electrospun PVA nanofibers. As shown in Figure 16e, PVA fiber swells with the increase of RH, resulting in the discrete surface area of the gold layer and the decrease of fiber conductivity. Thanks to the superior breathability of the substrate, the sensor can precisely monitor the humidity of skin and plants for a long time. To improve the anti‐interference of the humidity sensor, Zhang's group^[^ ^159^ ^]^ created a highly selective humidity sensor based on a PIM made of PVA/KOH polymer gel electrolyte as exhibited in Figure 16f. The PIM‐based sensor shows a fast and reversible response to humidity changes. As the RH increases from 10.89% to 81.75%, the conductance of PIM changes more than 70 times. Meanwhile, it is insensitive to changes in temperature (0–95 °C) and pressure (0–6.8 kPa).

#### 4.2.2 Chemical Sensors

**Figure 17:**
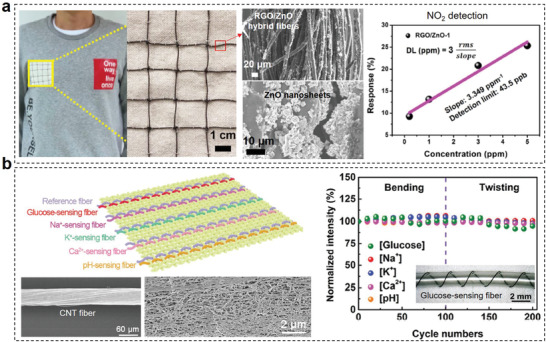
Chemical sensors based on flexible porous substrates. a) Wearable NO_2_ e‑textile gas sensor based on RGO/ZnO hybrid fibers with a low theoretical detection limit. Reproduced with permission.^[^ ^231^ ^]^ Copyright 2019, American Chemical Society. b) Multifunctional electrochemical fabric sensor based on CNT‐based sensing fibers with high working stability under repeated bending and twisting. Reproduced with permission.^[^ ^234^ ^]^ Copyright 2018, Wiley‐VCH.

Gas sensors can detect poisonous and flammable gaseous chemicals in the environment, such as NH_3_, NO_2_, ethanol, and acetaldehyde. Room‐temperature gas sensors have been successfully fabricated on diverse flexible porous substrates including fabrics,^[^ ^116^ ^]^ electrospun nanofibers,^[^ ^228^ ^]^ papers,^[^ ^229^ ^]^ and porous LCP.^[^ ^125^ ^]^ Owing to their high conductivity, large specific surface area, and strong mechanical property, carbon‐based materials are ideal sensing materials for gas sensors. To improve the selective adsorption of the sensing layer, metal oxides (ZnO)^[^ ^30^ ^]^ and conductive polymers (i.e., PPy and PANI)^[^ ^230^ ^]^ are usually coated on these carbon materials. Generally, when exposed to an analyte gas, the resulting sensor will exhibit a positive or negative conductance/resistance shift. The amount of shift is positively correlated with the gas concentration. The porous sensing layer and substrate can provide abundant adsorption sites, charge transport paths, and excellent deformability, which is beneficial to improve the sensitivity and stability of the sensor.^[^ ^125^ ^]^ **Figure** **17**a presents a wearable NO_2_ e‑textile gas sensor, using cotton/elastic threads coated with RGO/ZnO as sensing elements.^[^ ^231^ ^]^ The densely mesoporous structures of ZnO nanosheets provide a large contact space for the absorption and diffusion of NO_2_, resulting in a low detection limit (43.5 ppb). Due to the superior flexibility of the substrate, the sensor exhibits excellent long‐term stability (84 days), outstanding mechanical deformation tolerance (3000 bending cycles, 1000 twisting cycles, and 65% strain strength), and good washing durability (no noticeable response deterioration after repeated washing for eight times).

Chemical sensors can also detect physiological signals in liquid samples (i.e., blood serum, urine, and sweat), such as glucose, Na^+^, K^+^, Ca^2+^, pH, and DNA.^[^ ^23^ ^]^ Disposable electrochemical sensors based on paper and fabric have been extensively studied for the development of affordable point‐of‐care devices. For example, low‐cost electrochemical glucose sensors can be easily achieved by patterning functional materials (e.g., carbon,^[^ ^232^ ^]^ CNT, MWCNT,^[^ ^84^ ^]^ and NiSe_2_)^[^ ^233^ ^]^ immobilized with glucose oxidase (GOx) on paper/fabric. The electrochemical oxidation process of glucose is often recorded by the amperometric response curve. The glucose concentration can be derived by calculating the number of electrons involved in the electrooxidation process. By changing the sensing material, other physiological signals can be detected. Figure 17b demonstrates a multifunctional electrochemical fabric sensor manufactured by weaving CNT‐based sensing fibers with different functions.^[^ ^234^ ^]^ The resulting fabric sensor shows high flexibility and excellent working stability under repeated bending and twisting, and can efficiently detect glucose, Na^+^, K^+^, Ca^2+^, and pH.

### 4.3 Energy Storage and Conversion Devices

To meet the energy demands of electronic devices on flexible porous substrates, various energy storage and conversion devices have been invented. The typical energy storage devices are SCs and batteries, which can store electrical energy through different electrochemical processes. Nanogenerators (NGs) are recent notable sustainable energy conversion equipment, which can convert various environmental mechanical energy (e.g., human motions, water flows, winds, and acoustic waves) into electricity.

#### 4.3.1 Batteries and Supercapacitors

Both batteries and SCs consist of two electrodes separated by an electrolyte. Batteries store the electric charge in the form of chemical energy through electrochemical redox reactions, which can supply continuous high energy. SCs store the electrostatic charge at the interface between the electrolyte and the electrodes by forming an electric double layer, which is beneficial for applications that require fast charge/discharge and long‐cycle stability.

**Figure 18:**
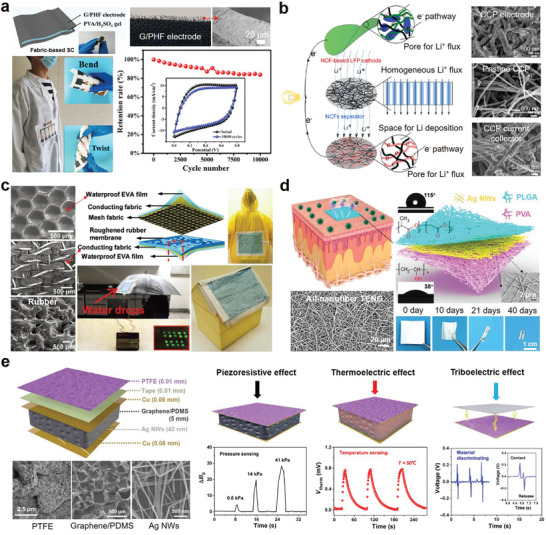
Energy storage and conversion devices based on flexible porous substrates. a) Multidimensional hierarchical bionic fabric‐based SC. Reproduced with permission.^[^ ^239^ ^]^ Copyright 2019, American Chemical Society. b) All‐nanocellulose‐based lithium metal batteries fabricated by integrating various functional materials into nanocellulose fibers. Reproduced with permission.^[^ ^246^ ^]^ Copyright 2018, American Chemical Society. c) Multifunctional TENGs composed of waterproof conductive fabric and roughened rubber membrane for multiple renewable energy harvesting. Reproduced with permission.^[^ ^255^ ^]^ Copyright 2019, Wiley‐VCH. d) All‐nanofiber TENG manufactured by sandwiching Ag NWs between electrospun PLGA and PVA nanofibers for biodegradable self‐powered e‐skin. Reproduced with permission.^[^ ^257^ ^]^ Copyright 2020, The Authors, some rights reserved; exclusive licensee American Association for the Advancement of Science. e) Multifunctional hybrid NG consisting of hydrophobic PTFE films and graphene/PDMS sponge to sense pressure, temperature, and material. Reproduced with permission.^[^ ^266^ ^]^ Copyright 2020, The Authors, some rights reserved; exclusive licensee American Association for the Advancement of Science.

To develop low‐cost batteries^[^ ^235^ ^]^ and SCs,^[^ ^6^ ^]^ flexible porous materials such as papers,^[^ ^236^ ^]^ fabrics,^[^ ^237^ ^]^ and electrospun nanofibers^[^ ^91^ ^]^ have been broadly used as their electrode, electrolyte, and substrate. Among them, cellulose‐based paper/fabrics have drawn much attention as biodegradable substrates for green energy storage devices. Electrochemical active cellulose‐based electrodes can be produced by integrating cellulose fibers with metals,^[^ ^185^ ^]^ carbon‐based materials,^[^ ^238^ ^]^ polymers (e.g., PEDOT,^[^ ^239^ ^]^ PANI),^[^ ^240^ ^]^ MXenes,^[^ ^241^ ^]^ or their composites.^[^ ^242^ ^]^ For instance, inspired by the sophisticated structures of hedgehog spines, Li et al.^[^ ^239^ ^]^ created a multidimensional hierarchical fabric‐based SC with enhanced energy storage performance. The bionic fiber microarray structure of the graphene/PEDOT‐coated hierarchical fabric (G/PHF) electrode is illustrated in **Figure** **18**a, which helps to load more electrochemical active materials. Hence, the assembled SC exhibits a high specific areal capacitance (245.5 mF cm^−2^ at 1 mV cm^−2^), an excellent energy density (21.82 μWh cm^−2^ at 0.4 mW cm^−2^), and a high capacitance retention ratio of 83.9% after 10 000 cycles. In addition, intrinsic conductive papers such as carbon fiber paper^[^ ^243^ ^]^ and MXene‐sulfur paper^[^ ^244^ ^]^ are also promising electrode materials, which have been used in vanadium flow batteries and Li‐S batteries.

Taking advantage of other properties of cellulose (e.g., capillarity, biocompatibility, and insulation), it can work as other functional components in energy storage devices. For example, using its capillarity, cellulose paper has been applied to absorb and store the electrolyte for paper‐based Al‐air batteries.^[^ ^63^ ^]^ With the help of its biocompatibility, Choi's group^[^ ^245^ ^]^ developed hybrid paper‐polymer microbial fuel cells which can biodegrade automatically in water without further treatment. The microporous, hydrophilic network of intertwined cellulose fibers in the paper enables rapid adsorption of bacterial cells and facilitates their accumulation. In addition, Wang et al.^[^ ^246^ ^]^ achieved all‐nanocellulose‐based lithium metal batteries by integrating various functional materials into nanocellulose fibers as shown in Figure 18b. Untreated nanocellulose fibers (NCFs) served as separators and conducting cellulose papers (CCPs) obtained by combining NCFs with carbon nanofibers acting as current collectors. Li electrodes were fabricated by electrodepositing Li on CCP, and positive electrodes are realized by embedding LiFePO_4_ (LFP) particles in CCP. The NCF separator with uniform pore distribution greatly improves the safety and lifespan of the battery because it brings a uniform current distribution at the electrode, which leads to a uniform lithium deposition on the negative electrode. Therefore, the battery demonstrates excellent cycling stability, with 85% capacity retention after 1000 cycles at a rate of 1.27 mA cm^−2^.

#### 4.3.2 Nanogenerators

NGs can harvest small‐scale energies in ambient environment and convert them into electricity.^[^ ^247^ ^]^ Piezoelectric nanogenerator (PENG) and TENG are two representative NGs.^[^ ^248^ ^]^ They can harvest low‐frequency mechanical energy from human motion, acoustic waves, water waves, rain, and wind.^[^ ^249^ ^]^ They can also function as self‐powered sensors for tactile sensing,^[^ ^250^ ^]^ human motion monitoring, and HMIs,^[^ ^251^ ^]^ because the current signal generated can reflect the type and magnitude of mechanical stimuli.

Porous materials such as papers,^[^ ^252^ ^]^ fabrics, and electrospun nanofibers^[^ ^253^ ^]^ have been widely used as the triboelectric layer, conductive layer, or substrate of TENGs. For example, by assembling copper‐coated multiholed paper with PTFE membrane, Fan et al.^[^ ^254^ ^]^ developed a paper‐based TENG for acoustic energy harvesting and self‐powered sound recording. Using waterproof ethylene‐vinyl acetate (EVA) film, conductive fabric, and roughened rubber membrane, Lai et al.^[^ ^255^ ^]^ designed multifunctional TENGs. As shown in Figure 18c, the fabric‐based TENG can be mounted on umbrellas, raincoats, and buildings to harvest energy from rains, winds, and various human movements. In addition, biodegradable electrospun nanofibers such as PVA^[^ ^256^ ^]^ and PLGA^[^ ^257^ ^]^ can be utilized to fabricate wearable TENGs with better biocompatibility, softness, and comfort. Figure 18d displays an all‐nanofiber TENG manufactured by sandwiching Ag NWs between electrospun PLGA and PVA nanofibers for breathable, biodegradable, antibacterial, and self‐powered e‐skin.^[^ ^257^ ^]^ These hierarchical porous nanofibers not only provide a high specific surface area for contact electrification but also offer numerous capillary channels for thermal‐moisture transfer. The fabricated e‐skin can monitor whole‐body physiological signals and joint movement in real‐time.

Elastic porous materials such as PDMS sponges^[^ ^258^ ^]^ are attractive substrates for highly compressible NGs due to their excellent deformability and recovery ability. For instance, Chun et al.^[^ ^259^ ^]^ impregnated gold nanoparticles in a mesoporous PDMS film as the compressible dielectric layer of TENG. Compared with flat film TENG, the dielectric constant of this porous PDMS increases more rapidly under the same mechanical force, which greatly improves the electrical output. The large surface area of the porous PDMS can increase the surface charge during the film contact, thereby further boosting the total output power. Natural porous materials such as wood sponge and natural polymers (e.g., cellulose, chitin, silk fibroin, rice paper, egg white)^[^ ^260^ ^]^ have been widely explored as substrates for biodegradable NGs. By treating wood sponge with a simple delignification process, Sun et al.^[^ ^261^ ^]^ proposed a low‐cost, biodegradable PENG. Compared with the original wood, the delignified wood (15 × 15 × 14 mm^3^) shows higher piezoelectric output (85 times higher, up to 0.69 V) due to its increased compressibility.

Other types of NG based on flexible porous substrates, including thermoelectric,^[^ ^262^ ^]^ moist‐electric,^[^ ^263^ ^]^ and near field communication‐based generators^[^ ^264^ ^]^ can collect energy from human body waste heat, moisture, and magnetic fields. In addition, hybrid NGs can be manufactured by integrating multiple electrical energy conversion materials. For example, applying nitrocellulose nanofibril paper as the triboelectric layer and BaTiO_3_/MWCNT@bacterial cellulose paper as the piezoelectric layer, Wang's group^[^ ^265^ ^]^ produced a paper‐based hybrid tribo/piezoelectric NG with integrated outputs of 18 V and 1.6 µA·cm^−2^. Recently, the same group^[^ ^266^ ^]^ designed a multifunctional hybrid NG consisting of hydrophobic PTFE films and graphene/PDMS sponges to sense pressure, temperature, and material. As shown in Figure 18e, the hybrid NG can detect temperature through thermoelectric effect (one‐kelvin resolution) and sense pressure through piezoresistive effect (sensitivity >15.22 kPa^−1^, response time <74 ms). Based on contact‐induced electrification, it can also distinguish different materials by generating distinct output voltage signals for various physical contacts.

### 4.4 Other Applications

#### 4.4.1 Actuators

**Figure 19:**
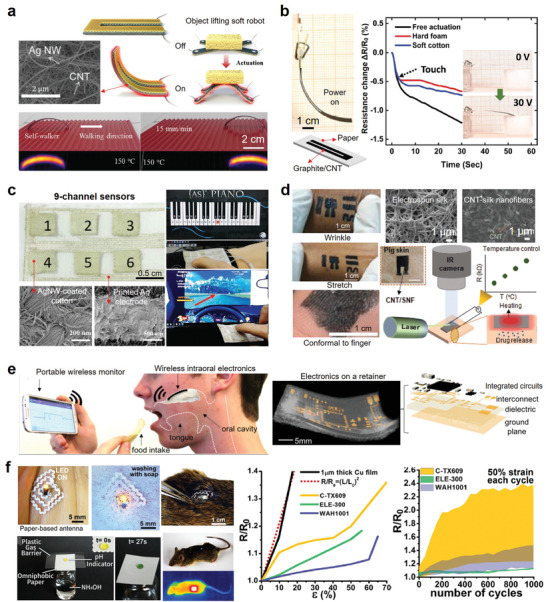
Actuator, HMI, and biomedical devices based on flexible porous substrates. a) Fabric‐based electrothermal actuator for biomimetic self‐walking robots and object lifting robots. Reproduced with permission.^[^ ^267^ ^]^ Copyright 2019, Wiley‐VCH. b) Self‐sensing paper actuator that can distinguish the touch of soft and hard objects based on different piezoresistive responses. Reproduced with permission.^[^ ^271^ ^]^ Copyright 2018, Wiley‐VCH. c) Fabric HMI device based on a piezoresistive sensor for piano and computer game playing. Reproduced with permission.^[^ ^274^ ^]^ Copyright 2018, Royal Society of Chemistry. d) Multifunctional epidermal E‐tattoos based on CNT/electrospun silk nanofibers. Reproduced with permission.^[^ ^279^ ^]^ Copyright 2021, Wiley‐VCH. e) Wireless, intraoral electronic devices for real‐time sodium intake monitoring toward hypertension management. Reproduced with permission.^[^ ^281^ ^]^ Copyright 2018, National Academy of Sciences. f) Implantable epidermal paper‐based devices for optogenetic stimulation and targeted cancer therapy showing excellent breathability, strong solderability of electronic components, and robust mechanical stability upon repetitive stretching. Reproduced with permission.^[^ ^282^ ^]^ Copyright 2018, American Chemical Society.

Electro‐active paper/fabric actuators that can bend, twist, and coil upon application of a voltage can be employed in soft robotics,^[^ ^267^ ^]^ artificial muscles,^[^ ^268^ ^]^ and biomimetic devices.^[^ ^269^ ^]^ Traditional ionic liquid impregnated paper actuators work based on a combination of ion migration and piezoelectric effect. For other electroactive paper/fabric actuators, electrothermal actuation is the most used working mechanism, and different movements can be achieved by designing the heat distribution of the heating layer. Carbon‐based materials (CNT,^[^ ^270^ ^]^ MWCNT,^[^ ^137^ ^]^ graphite)^[^ ^271^ ^]^ are the basic components of the heating layer. Adding metal nanoparticles (e.g., Ag, Au) can further improve the conductivity of the heating layer. **Figure** **19**a presents the fabric‐based electrothermal actuator reported by Ahn et al.^[^ ^267^ ^]^ for use in biomimetic self‐walking robots and object lifting robots. The conductive Ag NW/CNT composite heating layer can be driven by low voltage (<12 V). The porous fabric substrate not only helps to achieve a thick nanomaterial coating with high conductivity but also enables strong bonding between the actuator multilayers to maintain mechanical stability. Apart from performing actuation motions, the electrothermal actuator can implement sensing functions. Figure 19b exhibits a self‐sensing paper actuator, which can distinguish the touch of soft and hard objects based on different piezoresistive responses.^[^ ^271^ ^]^ The heating layer was fabricated by printing graphite‐CNT composite on cellulose paper. The porosity and hydrophilicity of the paper significantly enhance the ink adhesion, resulting in superior mechanical stability under bending, twisting, and even folding.

#### 4.4.2 Human–Machine Interfaces

The HMI helps people interact with machines, which is an indispensable part of the Internet of Things era. Generally, various sensors (e.g., pressure sensors, capacitive touch sensors, TENGs) constitute a fundamental interactive interface that perceives human behaviors and sends corresponding signals to the machine.^[^ ^272^ ^]^ Sensors based on flexible porous substrates (e.g., cellulose paper,^[^ ^273^ ^]^ fabric,^[^ ^274^ ^]^ leather,^[^ ^126^ ^]^ and porous PU film)^[^ ^275^ ^]^ are particularly suitable for wearable HMI applications because they are breathable, biocompatible, stretchable, and can conformally contact with the skin for a long time without side effects. Typical application demonstrations of HMI devices based on porous substrates include playing music and computer games,^[^ ^275^ ^]^ remote touching monitoring,^[^ ^276^ ^]^ e‐skin display,^[^ ^126^ ^]^ and self‐powered keypad.^[^ ^273^ ^]^ Figure 19c illustrates a fabric HMI based on a piezoresistive sensor for piano and computer game playing.^[^ ^274^ ^]^ The fabric HMI is assembled by sandwiching Ag NW‐coated cotton fabric between silver electrode printed cotton fabric and VHB tape. Owing to the large surface roughness and ultra‐low resistance of the functionalized fabric, the resulting HMI shows excellent detection performance, including high sensitivity in a wide pressure range (0–30 kPa), low detection limit (0.76 Pa), and fast response time (6 ms).

#### 4.4.3 Biomedical Devices

Wearable electronic devices that can continuously measure electrophysiological signals, store data, and provide feedback therapy and on‐demand drug delivery are a radical advancement in the field of personal healthcare. A variety of multifunctional wearable devices have been fabricated on flexible porous substrates (e.g., paper,^[^ ^277^ ^]^ fabric,^[^ ^278^ ^]^ electrospun silk nanofiber,^[^ ^279^ ^]^ porous polymeric substrate)^[^ ^280^ ^]^ for closed‐loop diagnosis and therapy applications. Their main components include physiological sensors, memory components, active heaters, temperature sensors, and drug‐release actuators. Physiological sensors can detect disease‐related biosignals such as the temperature of joints (gout),^[^ ^277^ ^]^ the ammonia concentration in breath (nephropathy),^[^ ^278^ ^]^ and motion‐related neurological disorders (movement disorders).^[^ ^280^ ^]^ These monitored data can be stored in integrated memory devices and categorized into specific disease modes. Then, the active heaters will implement the feedback therapy by triggering a thermal‐stimulated transdermal drug delivery. Simultaneously, the temperature sensor can monitor the skin temperature to prevent skin burns during thermotherapy. Figure 19d shows epidermal E‐tattoos based on CNT/electrospun silk nanofibers.^[^ ^279^ ^]^ They can be conformally attached to fingertips and forehands to monitor skin temperature, ECG, and EMG, and can also conduct thermotherapy and heat‐stimulated drug delivery.

In addition to on‐skin diagnostic and therapeutic applications, biocompatible/bioresorbable biomedical devices can be inserted in the oral cavity or implanted in vivo for health monitoring and targeted therapy. For example, based on an ultrasoft microporous membrane, Lee et al.^[^ ^281^ ^]^ generated a wireless intraoral electronic device for real‐time sodium intake monitoring toward hypertension management. As shown in Figure 19e, the device consists of chip‐scale components (sodium sensors, stretchable interconnects, microporous membrane) that can be laminated on a custom‐made porous retainer. The open mesh multimembrane structure of the device makes it easily integrated with any type of flexible, foldable, and movable fixtures for body wearing or insertion. The retainer made of low modulus microporous elastomer provides great comfort in contact with oral tissue. Figure 19f demonstrates implantable epidermal paper‐based devices proposed by Sadri et al.^[^ ^282^ ^]^ for optogenetic stimulation and targeted cancer therapy through Joule heating. The device is easy to manufacture by laser cutting the Ag nanoflake‐coated paper and shows excellent breathability which allows the permeation of NH_3_ gas and reacts with the pH indicator. The omniphobic paper substrate also can protect the conductive Ag nanoflake layer from oxidizing underwater during washing. After the encapsulation in a biocompatible PDMS, the paper‐based antenna can be implanted in vivo, making it useful for optogenetic stimulation. In addition, the fibrous paper substrate not only facilitates the attachment of electronic components but also accommodates deformation during stretching and prevents the propagation of cracks on the device, leading to strong solderability of electronic components, and robust mechanical stability upon repetitive stretching. As displayed in the normalized resistance–strain curves, the device fabricated in 100% cellulose paper (ELE‐300) can withstand a large deformation (*ε*_max_ up to 68%) before breaking and maintain a good electrical performance after repeated straining of 50% over 1000 stretch–release cycles.

## 5 Challenges and Outlook

The outstanding advantages of flexible porous substrates over traditional plastic or metal substrates are light weight, large specific surface area, biocompatibility, and strong deformability. These merits make them attractive substrates for new‐generation electronic devices that are breathable, wearable, implantable, and stretchable. By integrating active/conductive materials on porous substrates through different functionalization methods, their electronic applications can be expanded from basic circuits to hybrid multifunctional biomedical devices that integrate sensors, memory components, heaters, actuators, and energy supply components. Despite these tremendous innovations and progress of electronic devices on flexible porous substrates, there are still some issues to be addressed.

1. Advanced materials: At present, both the raw materials for porous substrate fabrication and active nanomaterials for its functionalization are limited and have certain applicable conditions. On one hand, new synthetic materials should be developed to produce porous substrates with desirable porosity, biocompatibility, deformability, and mechanical strength. New active nanomaterials need to be investigated to help the porous substrate achieve high conductivity and other novel functions. On the other hand, nature provides us with endless creative inspiration. We can refer to natural porous materials to make artificial bionic materials or explore more natural porous materials that can be used for electronic devices.
2. Efficient fabrication approaches: Although the reported prototypes of electronic devices based on flexible porous substrates show good prospects, these devices are still in the early stage of commercialization. In addition to cheap substrates, there is an urgent need for efficient, simple, cost‐effective, industrial scalable, and green manufacturing technologies to develop more practical electronic devices on flexible porous substrates.
3. Optimized device performance: The porosity and surface roughness of flexible porous substrates can improve the adhesion of active materials and the performance of sensors and energy devices. However, compared with plastic substrates, they typically result in lower conductivity of printed conducting materials and are unsuitable for the preparation of organic electronics (e.g., organic diodes, transistors, solar cells). It is necessary to optimize the porous structure and conduct in‐depth studies on interfacial properties to further improve the processing compatibility of flexible porous substrates and promote device performance (e.g., conductivity, fatigue strength, damage tolerance).
4. Better user experience: The current electronic devices based on flexible porous substrates have considered a certain degree of multi‐functional integration, portability, and wearing comfort. The user experience can be further improved in the following aspects: i) All materials used in electronic devices, including substrates and conductive/active materials, should be highly biocompatible and safe to avoid any side effects on humans and the environment. ii) Wireless, self‐powered, durable, and all‐in‐one electronic devices that can be seamlessly integrated with clothes/skin or can be implanted in vivo will be in high demand in the future. iii) With the further penetration of electronic devices in all aspects of human life, future electronic devices need to ensure the security of private information.

Shortly, electronic devices based on flexible porous substrates are anticipated to speed up the evolution of a new generation of low‐cost, high‐performance personal health devices, HMIs, implantable electronics, and soft robots, but at present, numerous research works are still needed to transform laboratory prototype devices into commercialized products. First, further progress is needed in the design and control of pore architectures of the porous substrate because the pore structures (e.g., shape, size, distribution, and connectivity) directly affect the properties of the substrate (e.g., surface area, density, permeability, softness, deformability, and load‐bearing property), thereby influencing the subsequent manufacturing process, and resulting in different device performance. In the future, integrating 3D printing into the production of porous substrates may be a promising method for achieving more precise control of pore structures. Second, it is necessary to establish a deeper theoretical basis for porous substrates, including their physical properties, chemical composition, and porous structure‐device performance relationship. Due to the diversity and complexity of porous substrates, these new insights can effectively guide the optimization of the porous structure to better meet the needs of various applications. Last, multidisciplinary cooperation can vigorously promote the development of porous substrate‐based electronic devices, because this field involves material science, physical and chemical processes, electronic engineering, surface chemistry, solid mechanics, etc. In summary, flexible porous substrates are ubiquitous in our daily life, offering irreplaceable opportunities to seamlessly integrate electronic devices into all aspects of human clothing, food, housing, and transportation. Although their unique porous structure poses challenges to some applications, after overcoming the remaining challenges, we will usher in a new electronic era that makes our life more convenient.

## Conflict of Interest

The authors declare no conflict of interest.
